# MUTE drives asymmetric divisions to form stomatal subsidiary cells in Crassulaceae succulents

**DOI:** 10.1126/sciadv.aeb8145

**Published:** 2026-03-25

**Authors:** Xin Cheng, Heike Lindner, Lidia Hoffmann, Antonio Aristides Pereira Gomes Filho, Paola Ruiz Duarte, Susanna F. Boxall, Yiğit Berkay Gündoğmuş, Jessica H. Pritchard, Sam Haldenby, Matthew Gemmell, Alistair Darby, Miro Läderach, James Hartwell, Michael T. Raissig

**Affiliations:** ^1^ Institute of Plant Sciences, University of Bern, 3013 Bern, Switzerland.; ^2^ Centre for Organismal Studies Heidelberg, Heidelberg University, 69120 Heidelberg, Germany.; ^3^ Oeschger Centre for Climate Change Research, University of Bern, 3012 Bern, Switzerland.; ^4^ Department of Biochemistry, Cell and Systems Biology, Institute of Systems, Molecular and Integrative Biology, University of Liverpool, Liverpool L69 7ZB, UK.

## Abstract

Among the evolutionary innovations of many succulents is a photosynthetic lifestyle, where stomatal gas exchange is decoupled from light-dependent carbon fixation. Many Crassulaceae leaf succulents form a stomatal morphotype consisting of kidney-shaped guard cells surrounded by three anisocytic subsidiary cells (SCs), whose function and development remained unknown. Here, we established *Kalanchoë laxiflora* as a developmental model. Potassium staining suggested SCs to shuttle osmolytes and support turgor-driven stomatal movements. Gene editing, reporter lines, protein overexpression, and RNA sequencing implicated the stomatal transcription factor *MUTE* in facilitating the additional rounds of asymmetric divisions required to form SCs in succulents. This is opposite to the role of *MUTE* in *Arabidopsis thaliana*, where it stops rather than induces asymmetric divisions but reminiscent of *MUTE*’s subsidiary cell–related function in grasses. Our work firmly establishes *K. laxiflora* as a model for succulent development and deciphers an intricate genetic mechanism that generates innovative stomatal morphology in Crassulaceae succulents.

## Teaser

Crassulaceae MUTE guides asymmetric divisions to form stomatal subsidiary cells in succulents.

## INTRODUCTION

**Fig. 1.**
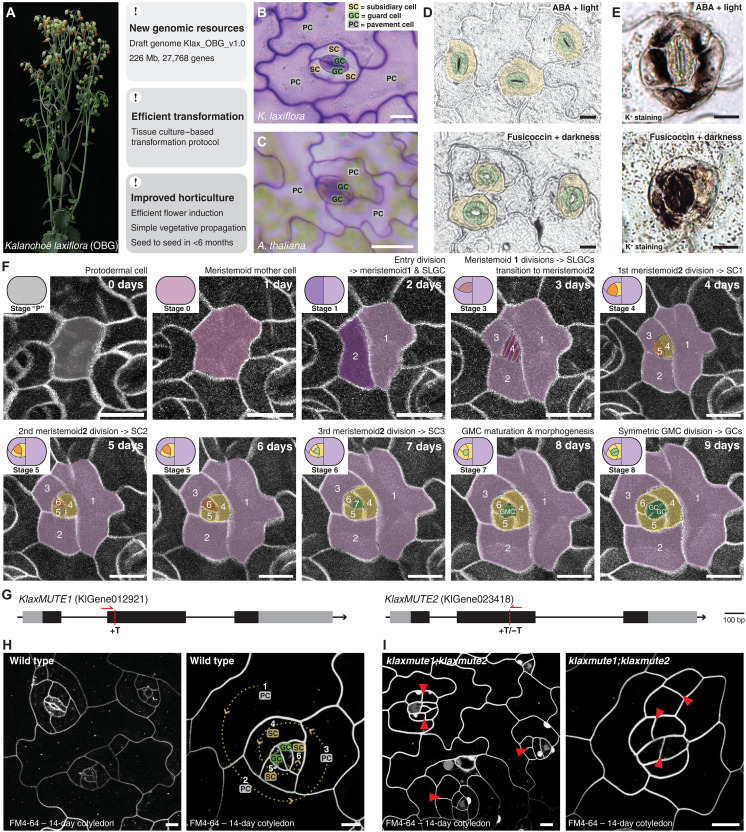
*MUTE*-dependent amplifying divisions form functionally relevant SCs in *K. laxiflora*. (**A**) Flowering *K. laxiflora*, an emerging succulent model plant. Freshly generated genomic resources, biotechnology, and horticultural protocols are indicated. (**B**) *K. laxiflora* stomata consisting of two central GCs surrounded by three anisocytic SCs. (**C**) Two-celled stomata of *A. thaliana* without SCs. (**D**) Abscisic acid (ABA; top) and Fusicoccin treatment (bottom) close and open stomata, respectively. GCs are false-colored in green and SCs in yellow. Scale bars, 20 μm. (**E**) Potassium (K^+^) staining shows K^+^ in SCs when stomata are closed and in GCs when stomata are open. Scale bars, 20 μm. (**F**) Ten-day time-lapse imaging of plasma membrane marker line (*35Sp:mCherry-AtPIP1;4*) reveals two distinct phases of spiraling asymmetric divisions; the first meristemoid1 (purple) phase forms three stomatal lineage ground cells (SLGCs; lilac). The second meristemoid2 (orange) phase forms three SCs (yellow). Then, the GMCs (green) divides symmetrically to form two GCs (green). Stages and days are indicated. Scale bars, 20 μm. (**G**) Gene models of the two duplicated *KlaxMUTE* homologs; gene editing sites and guide RNAs are indicated. (**H** and **I**) Developing 14-day-old cotyledons in wild type (H) and *klaxmute1;klaxmute2* double mutants (I). Fibonacci division spiral is indicated (yellow spiral; H), and ectopic, transverse divisions are indicated [red arrowheads; (I)]. Cell membranes visualized with FM4-64. Scale bars, 10 μm.

Land plants evolved numerous anatomical and physiological features to thrive under harsh terrestrial conditions. Among them are sealed leaves preventing desiccation, on which adjustable breathing pores (= stomata) are interspersed that enable plant-atmosphere gas exchange (*1*). Additional evolutionary innovations like plant organ succulence and Crassulacean acid metabolism (CAM), a highly water-use efficient photosynthetic lifestyle, further unlocked extremely water-scarce environments (*2*–*4*). CAM plants open stomata primarily at night and fix carbon dioxide (CO_2_) into an organic acid intermediate stored in vacuoles. During the day, stomata are mostly closed, the storage intermediate is decarboxylated, and the released CO_2_ is assimilated in the Calvin-Benson-Bassham cycle (*5*, *6*). Most succulent plants use CAM photosynthesis, and many CAM plants form stomata, where the central guard cells (GCs) are flanked or surrounded by putative subsidiary cells (SCs) (*7*). Stomata in the leaf succulent *Kalanchoë laxiflora* (*8*, *9*), for example, consist of two kidney-shaped GCs surrounded by three unequally sized (= anisocytic), circularly arranged SCs (Fig. 1B). These SCs are distinct in size and shape compared to the puzzle-shaped pavement cells (PCs) and thus fulfill the original definition of stomatal SCs (*10*). Yet, although SCs are overrepresented in CAM plants, it remained unclear whether they serve as helper cells to support stomatal opening and closing like in grasses (*11*–*13*).

Unlike many CAM plants, the C_3_ eudicot and Brassicaceae model *Arabidopsis thaliana* does not form SCs (Fig. 1C). Consequently, the two GCs are surrounded by puzzle-shaped PCs only (Fig. 1C). In *A. thaliana*, three basic helix-loop-helix (bHLH) transcription factors guide stomatal development. *AtSPEECHLESS* (*AtSPCH*) determines stomatal precursors (= meristemoids) and drives asymmetric divisions (*14*). *AtMUTE* ends asymmetric divisions, establishes the guard mother cell (GMC), and induces the symmetric division forming the GC pair (*15*–*17*). Last, *AtFAMA* represses symmetric division potential and controls GC differentiation (*18*). While this bHLH module is conserved from mosses to flowering plants (*19*), it was amplified, subfunctionalized, modified, and rewired to generate different stomatal morphologies (*20*, *21*). In grasses, for example, MUTE is not required for GMC identity but rather acquired cell-to-cell mobility to establish subsidiary mother cells in cells adjacent to the GMC, which then divide asymmetrically to contribute perigene SCs (i.e., from a distinct cell lineage to those that lead to GCs) (*11*, *22*, *23*). In *K. laxiflora*, though, both SCs and GCs were suggested to be formed by the same precursor cell (= meristemoid), which seems to undergo many rounds of spiraling amplifying divisions, resulting in mesogene SCs (i.e., from the same lineage as GCs) (*24*, *25*). Yet how often, and in which pattern, meristemoids divide, why some divisions yield SCs and which genetic programs control these divisions remained elusive.

Here, we established the eudicot succulent CAM species *K. laxiflora* [Oxford Botanical Garden (OBG) accession] as a developmental model to determine the functional relevance of SCs, the division patterns that form SCs and the genetic program that enables SC divisions. We sequenced *K. laxiflora* OBG’s diploid genome, established efficient transgenesis protocols, and optimized horticultural protocols to efficiently induce flowering and viable seed production and reduce the life cycle to less than 6 months. Potassium (K^+^) staining revealed K^+^ shuttling between GCs and SCs during stomatal movements, suggesting that the SCs are physiological helper cells, as in grasses (*26*). Time-lapse imaging of developing leaves showed that additional rounds of amplifying divisions, which do not occur in *A. thaliana*, generated the mesogenous, anisocytic SCs. Mutant, reporter, and overexpression analysis of the duplicated *KlaxMUTE1* and *KlaxMUTE2* genes suggested that *Kalanchoë* MUTEs control an asymmetric division program that generates SCs. *KlaxMUTEs*’ function in driving SC divisions was species context dependent and could not be transferred to *A. thaliana*. Last, transcriptome analysis indicated that *KlaxMUTE1* activated specific cell cycle programs associated with asymmetric rather than symmetric cell divisions and delayed GC differentiation. Together, our work uncovered the intricate genetic mechanisms that govern the formation of functionally relevant, anisocytic SCs in the Crassulaceae family, which might be adaptive to the CAM photosynthetic lifestyle based on their abundance in CAM lineages (*7*).

## RESULTS

### A previously unknown model system for succulent development

The *Kalanchoë* genus is used since more than a decade to experimentally study molecular aspects of CAM in *Kalanchoë fedtschenkoi* (*8*) or leaf plantlet formation in *Kalanchoë daigremontiana* (*27*, *28*). The major disadvantage of these two species, however, is inefficient flower induction and the formation of nonviable seeds causing long generation times and preventing the combination of genotypes through crossings. We therefore established the diploid *K. laxiflora* OBG accession as a fast and fertile experimental, genetic model system (Fig. 1A). We generated a draft genome of *K. laxiflora* OBG consisting of 226 Mb in 78 contigs with 27,768 genes (Klax_OBG_v1.0, Fig. 1A, https://zenodo.org/records/14563707). We further streamlined a highly efficient tissue culture–based transformation protocol and established several essential horticultural protocols like flower induction, seed production, crossing, and vegetative propagation. Last, we optimized *K. laxiflora*’s seed-to-seed life cycle to <6 months (Fig. 1A). Together, this firmly established *K. laxiflora* OBG as a readily transformable and genetically accessible succulent developmental model system.

### K^+^ shuttling during stomatal movements suggests SCs to be functionally relevant

To determine whether *Kalanchoë* SCs are helper cells that contribute to stomatal opening and closing, we precipitated the major stomatal osmolyte potassium (K^+^) in open and closed stomata in *K. laxiflora*. Incubation of epidermal leaf peels with Fusicoccin and abscisic acid (ABA) successfully opened and closed stomata, respectively (Fig. 1D). Upon treatments, K^+^ precipitation showed that K^+^ primarily resides in SCs in closed stomata and moves to GCs in open stomata (Fig. 1E). This strongly suggests that, much like in grasses, *K. laxiflora* SCs contribute to osmolyte shuttling and thus reciprocal turgor control of stomatal cells (*26*). Quantification of K^+^ levels in open and closed stomata, however, suggested that residual K^+^ remains in SCs even when stomata are open (fig. S1, A and B). Although K^+^ is also shuttled between PCs and GCs in *A. thaliana* (fig. S1C), the substantial size difference between PCs and GCs makes reciprocal turgor regulation between these two cell types rather unlikely. In summary, we propose that *K. laxiflora* forms stomatal SCs characterized by their distinct shape, size, and arrangement (*10*) and the reciprocal K^+^ shuttling between similarly sized cells potentially enabling reciprocal turgor regulation between GCs and SCs.

### Additional rounds of asymmetric meristemoid divisions generate SCs

Mature *Kalanchoë* stomata consist of two paired GCs surrounding the pore and three unequally sized and circularly arranged SCs. In addition, the complete stomatal complex is surrounded by two to three PCs (Fig. 1B). We previously suggested that protodermal cells undergo five to six rounds of stem cell–like asymmetric cell divisions (ACDs) following a Fibonacci spiral pattern, where the first two to three divisions generate stomatal lineage ground cells (SLGCs) and the following three ACDs generate SCs (*25*). To unequivocally describe stomatal development in *K. laxiflora*, we used manual time-lapse imaging of developing leaves expressing a plasma membrane reporter gene (*35Sp:mCherry-AtPIP1;4*) to follow developing stomatal complexes over 10 days (Fig. 1F and movie S1). A meristemoid mother cell executed an initial entry division (stage 1; Fig. 1F). The smaller cell then divided asymmetrically two more times forming SLGCs while maintaining a small, stem cell–like meristemoid (stage 3; Fig. 1F). Much like in *A. thaliana*, SLGCs can directly differentiate into PCs or execute a spacing division to establish an additional meristemoid (fig. S2 and movie S2). After the entry division and two SLGC divisions, the meristemoid divided three additional times to produce SCs, which could not execute further spacing divisions (stages 4 to 6; Fig. 1F). After three SC divisions, the meristemoid then adopted GMC identity, rounded up (stage 7; Fig. 1F), and divided symmetrically to form GCs (stage 8; Fig. 1F). We therefore suggest two distinct meristemoid stages: a meristemoid1 stage that produces SLGCs and a meristemoid2 stage that produces SCs (Fig. 1F). However, the developmental mechanisms and genetic modules that control the meristemoid1-to-meristemoid2 transition and the SLGC versus SC divisions remained unknown.

### *klaxmute1;klaxmute2* cannot form functional stomatal complexes and shows aberrant asymmetric divisions

In *A. thaliana*, the *MUTE* transcription factor ends meristemoid ACDs and controls the meristemoid-to-GMC transition (*15*–*17*). In grasses, however, *MUTE* controls SC formation and GMC division orientation (*11*, *22*, *29*). In *K. laxiflora*, *MUTE* is duplicated (*KlaxMUTE1* and *KlaxMUTE2*), and like in grasses, these MUTE homologs featured an extended, yet poorly conserved C-terminal amino acid tail (fig. S3). We used gene editing to induce mutations in both *KlaxMUTE* homologs (Fig. 1G), yet the single mutants developed normal stomata (fig. S4A). When crossed, however, *klaxmute1;klaxmute2* double mutants arrested growth as young seedlings and failed to form mature stomata (Fig. 1, H and I, and fig. S4B) reminiscent of the *atmute* phenotype in *A. thaliana* (*17*). In *klaxmute1;klaxmute2*, however, we observed ectopic transverse or misoriented divisions that violated the Fibonacci spiral pattern (Fig. 1I). Such ectopic, misoriented divisions are not found in *atmute*, where the extensive amplifying divisions do not violate the spiraling Fibonacci pattern (*17*). Together, this indicated an additional role for the *KlaxMUTEs* beyond controlling the meristemoid-to-GMC transition as observed in *A. thaliana*.

### *KlaxMUTE1* and *KlaxMUTE2* reporter genes are expressed during SC divisions

**Fig. 2.**
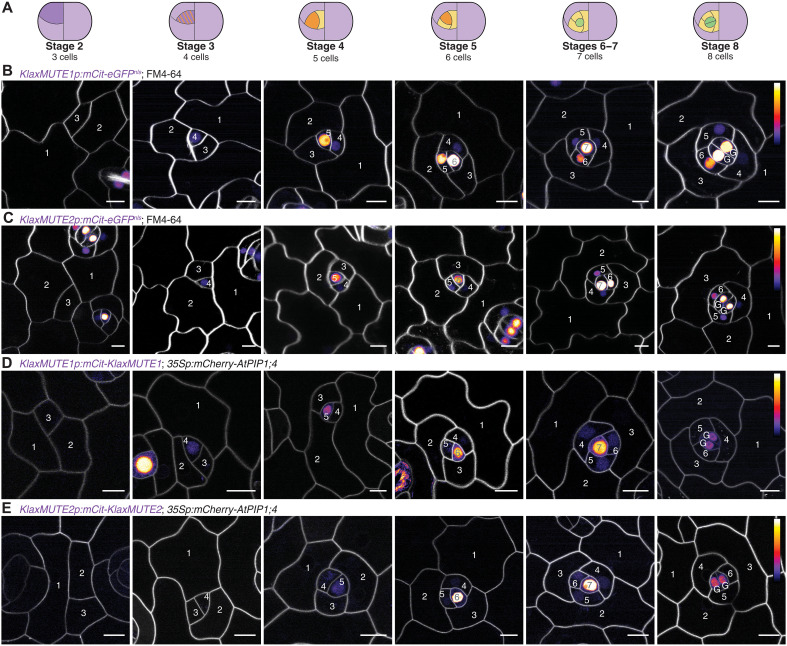
*KlaxMUTE1* and *KlaxMUTE2* are expressed throughout SC divisions. (**A**) Different developmental stages of stomata (stage 2 until stage 8) as shown below. (**B** and **C**) Confocal microscopy images of transcriptional reporters *KlaxMUTE1p:mCitrine-eGFP^nls^* (B) and *KlaxMUTE2p:mCitrine-eGFP^nls^* (C). Cell membranes (gray) were stained with FM4-64. Note that the leftmost and rightmost images of (C) show different sections of the same image source and slightly overlap. (**D** and **E**) Confocal microscopy images of translational reporters *KlaxMUTE1p:mCitrine-KlaxMUTE1* (D) and *KlaxMUTE2p:mCitrine-KlaxMUTE2* (E) also expressing the plasma membrane marker *35Sp:mCherry-AtPIP1;4* (in gray). Yellow fluorescent protein signal shown as fire intensity heatmap with a calibration bar indicated in the right most. Scale bars, 10 μm.

To observe when and where *KlaxMUTE1* and *KlaxMUTE2* are expressed, we generated transcriptional and translational reporter lines for both *KlaxMUTE* homologs. Notably, both transcriptional reporters (*KlaxMUTE1p:mCit-eGFP^nls^* and *KlaxMUTE2p:mCit-eGFP^nls^*) started to be weakly expressed in meristemoids after the first three ACDs (i.e., entry division and two SLGC divisions) concomitant with the meristemoid1-to-meristemoid2 transition (stage 3; Fig. 2, A to C). The two *KlaxMUTE* promoters remained active throughout all meristemoid2 divisions that generate SCs and peaked in GMCs (stages 4 to 7; Fig. 2, B and C). A similar expression window was observed with translational reporters (*KlaxMUTE1p:mCit-KlaxMUTE1*, and *KlaxMUTE2p:mCit-KlaxMUTE2*; Fig. 2, D and E). Very weak expression of the *KlaxMUTE1* translational reporter lines was first detected during the putative meristemoid1-to-meristemoid2 transition (stage 3) and was maintained until after the symmetric GMC division (stage 8; Fig. 2D). *KlaxMUTE1* seemed to be expressed earlier than *KlaxMUTE2*, which was not visible during the putative meristemoid1-to-meristemoid2 transition (Fig. 2E). During GMC formation, we observed strong nuclear and weak cytoplasmic signals in GMCs and a reappearing signal in SCs (stages 6 and 7; Fig. 2, D and E). The signal intensity decreased quickly in all cells after the symmetric GMC division (stage 8; Fig. 2, D and E) but remained longer in divided GCs than SCs, yet sometimes weak signal remained in the smallest SC (fig. S5, D and E). Together, the expression window of *KlaxMUTEs* coincided with SC divisions, suggesting a previously unknown role that facilitates the formation of anisocytic SCs.

### Time-lapse imaging of translational reporter lines confirms KlaxMUTE1 and KlaxMUTE2 expression during asymmetric divisions

**Fig. 3.**
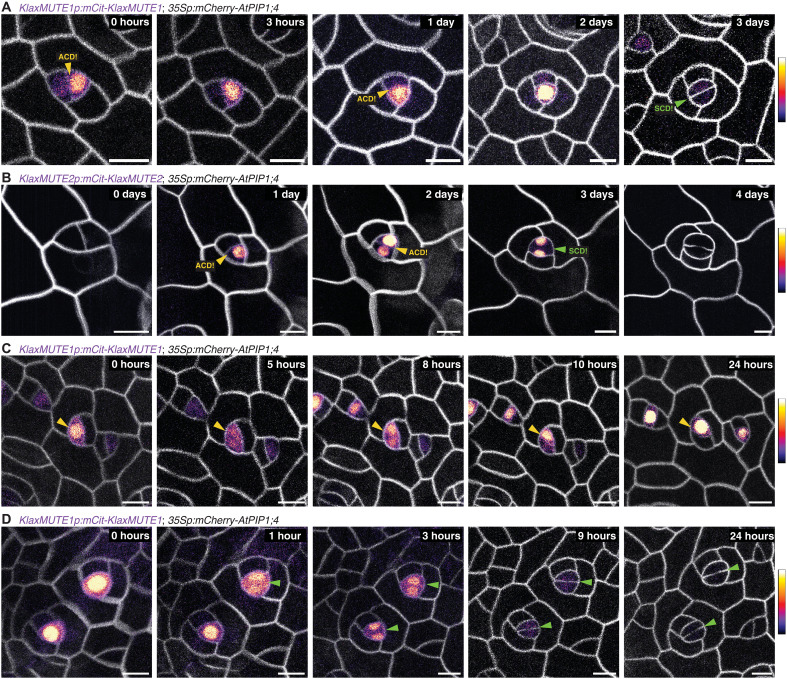
*KlaxMUTE1* and *KlaxMUTE2* dynamics during stomatal divisions. (**A** and **B**) Time-lapse confocal microscopy imaging of the translational reporters *KlaxMUTE1p:mCitrine-KlaxMUTE1* (A) and *KlaxMUTE2p:mCitrine-KlaxMUTE2* (B) with introgressed plasma membrane marker *35Sp:mCherry-AtPIP1;4.* (**C** and **D**) Dynamics of KlaxMUTE1 protein abundance during an asymmetric SC division (C) and symmetric GMC divisions (D). ACDs are highlighted with a yellow arrowhead, whereas symmetric cell divisions (SCDs) are highlighted with green arrowheads. The mCitrine signal is shown in a fire heatmap (see the heatmap scale bar on the right), and mCherry plasma membrane signal is shown in gray. Time intervals are indicated. Scale bar, 10 μm.

A limitation of single time point reporter imaging is that developmental stages are merely inferred from cell morphologies and Fibonacci-patterned cell divisions. Although these are relatively straightforward to retrace, the developmental stages are deductive. To follow *KlaxMUTE1* and *KlaxMUTE2* translational reporter expression over time in a single developing complex, we introgressed the plasma membrane marker line (*35Sp:mCherry-AtPIP1;4*) into the respective reporter lines. Time-lapse imaging over 5 consecutive days confirmed that both KlaxMUTE1 and KlaxMUTE2 are expressed before, during, and after asymmetric divisions that form SCs in addition to being expressed in the GMC before and during the symmetric division (Fig. 3, A and B). Despite using a less light-sensitive objective due to the time-lapse imaging setup than for the single time point imaging (Fig. 2), we found that KlaxMUTE1 was expressed for at least two rounds of ACDs (Fig. 3A), whereas KlaxMUTE2 was expressed for at least one SC-forming ACD confirming the slightly later and weaker expression of KlaxMUTE2 as suggested before (Fig. 2E). We then followed KlaxMUTE1 expression dynamics during a single ACD with a higher time resolution (one image per hour). We found that KlaxMUTE1 was strongly expressed in a meristemoid2 predivision and was present in both daughter cells postdivision. It was then quickly down-regulated in the future SC, whereas expression in the meristemoid2 was maintained (Fig. 3C). In GMCs, KlaxMUTE1 was strongly expressed before the symmetric division and then quickly down-regulated in both GCs postdivision (Fig. 3D). While the GMC expression dynamic is reminiscent of AtMUTE’s expression dynamic during GMC division in *A. thaliana*, the strong expression during SC-forming ACDs stood in stark contrast to *AtMUTE*’s role in ending asymmetric divisions in *A. thaliana*.

### Overexpression of *KlaxMUTEs* induces ectopic asymmetric divisions

**Fig. 4.**
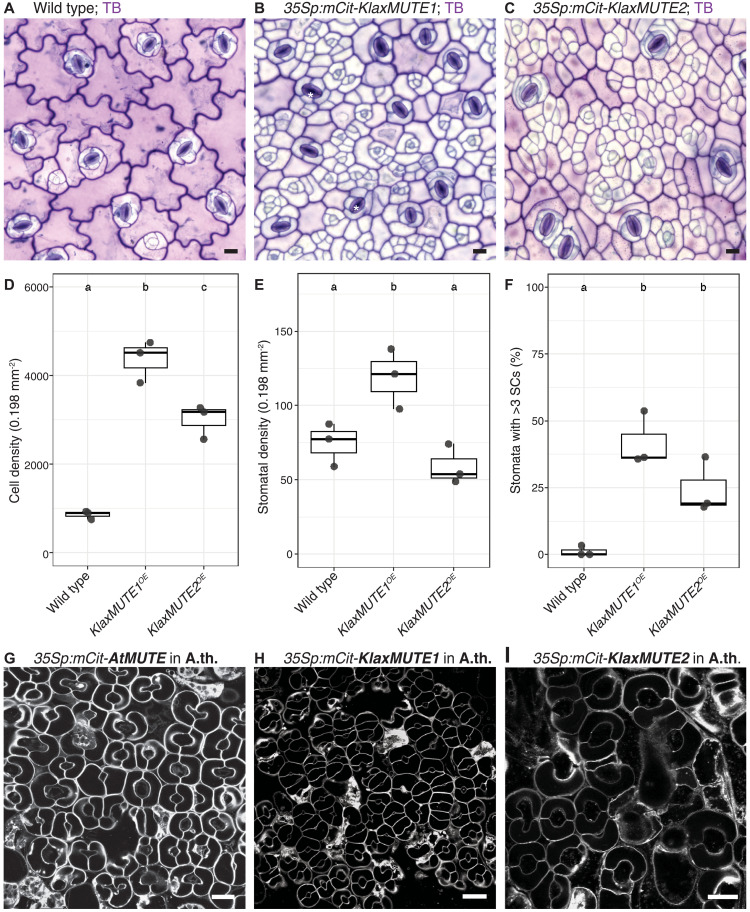
Overexpression of *KlaxMUTE1* and *KlaxMUTE2* induces ectopic asymmetric divisions in *K. laxiflora*. (**A** to **C**) Brightfield images of Toluidine Blue (TB)–stained epidermal peels of mature *K. laxiflora* leaves in wild type (A), *35Sp:mCitrine-KlaxMUTE1* (B), and *35S:mCitrine-KlaxMUTE2* (C). Scale bars, 20 μm. (**D** to **F**) Quantification of *KlaxMUTE1* and *KlaxMUTE2* overexpression phenotypes; cell density (D), stomatal density (E), and fraction of stomatal complexes with more than three SCs (F). Counted were three individuals per genotype, 1500 to 7780 total cells, and 100 to 212 stomata per genotype. One-way analysis of variance (ANOVA) followed by Tukey post hoc test, letters indicate significance groups. (**G** to **I**) Confocal images of FM4-64–stained cotyledons of *MUTE* overexpression lines in *A. thaliana* (A.th.); *35Sp:mCitrine-AtMUTE* (G), *35Sp:mCitrine-KlaxMUTE1* (H), and *35Sp:mCitrine-KlaxMUTE2* (I). Scale bars, 20 μm.

The key experiment in *A. thaliana* that firmly determined MUTE’s role in ending ACDs and committing GMC identity was its overexpression. Overexpressing *AtMUTE* produced a leaf epidermis solely consisting of paired GCs (*17*). However, overexpressing *KlaxMUTE1* (*35Sp:mCit-KlaxMUTE1 = KlaxMUTE1^OE^*) or *KlaxMUTE2* (*35Sp:mCit-KlaxMUTE2 = KlaxMUTE2^OE^*) in *K. laxiflora* caused a severe overabundance of small cells in the mature leaf epidermis and a notable absence of puzzle-shaped PCs (Fig. 4, A to C). The total cell density was increased fourfold and threefold in *KlaxMUTE1^OE^* and *KlaxMUTE2^OE^*, respectively (Fig. 4D and table S2). The stomatal density, on the other hand, was increased in *KlaxMUTE1^OE^* only and slightly and nonsignificantly decreased in *KlaxMUTE2^OE^* (Fig. 4E and table S2). Furthermore, more than 35 and 20% of stomatal complexes formed more than three SCs in *KlaxMUTE1^OE^* and *KlaxMUTE2^OE^*, respectively (Fig. 4F and table S2). Together, this strongly suggested that the *KlaxMUTEs* induce an asymmetric division program to form SCs rather than establishing GMC identity and ending ACDs, as is the case in *A. thaliana* (*17*).

Observing young developing leaves (0.5 cm) showed numerous ectopic ACDs and many more, and much smaller, cells in the leaf epidermis compared to similar-sized wild-type leaves (fig. S6, A to C). Quantification of postdivision cell size ratios in these young developing leaves in both *KlaxMUTE1^OE^* and *KlaxMUTE2^OE^* lines suggested that the physical asymmetry of divisions is stronger when overexpressing *KlaxMUTE1* compared to overexpressing *KlaxMUTE2* (fig. S6, D to F). This suggested a putative difference and weak subfunctionalization of *KlaxMUTE1* and *KlaxMUTE2*, although the single mutants did not show a mutant phenotype and the expression windows only slightly differ between the two genes. Nonetheless and despite these massive changes to leaf epidermal development in both overexpression lines, these plants grew reasonably well but formed slightly cup-shaped, curved leaves, which might be caused by imbalanced abaxial and adaxial tissue growth patterns as a consequence of ectopic divisions (fig. S7A).

While we cannot fully determine the identity of the ectopically formed small cells, circumstantial evidence suggested them to be SC-like cells. First, in Toluidine blue-stained epidermal peels of mature wild-type leaves, the three epidermal cell types have very specific staining patterns; GCs were purple, SCs were white (i.e., unstained), and PCs were pink (Fig. 4A). The numerous ectopic small cells in the overexpression lines mostly remained white indicating SC-like characteristics (Fig. 4, B and C). Furthermore, PCs are known to endoreduplicate and are thus of higher-order ploidies (*30*). Ploidy analysis of epidermal nuclei (i.e., of isolated peels) indicated fewer polyploid nuclei in the overexpression lines compared to wild type, further suggesting a smaller fraction of PCs and a larger fraction of SC-like cells (fig. S8).

Together, we conclude that the Crassulaceae *MUTEs* promote additional rounds of asymmetric, amplifying divisions required to form anisocytic SCs.

### The functional role of *KlaxMUTEs* is species-context dependent

Next, we wanted to test whether the ACD-promoting functionality of KlaxMUTE1 and KlaxMUTE2 was intrinsic to the protein and could thus be transferred to *A. thaliana*. However, overexpression of *AtMUTE* (*35Sp:mCit-AtMUTE*), *KlaxMUTE1* (*35Sp:mCit-KlaxMUTE1*), or *KlaxMUTE2* (*35Sp:mCit-KlaxMUTE2*) in *A. thaliana* solely induced paired GC formation and not ectopic ACDs (Fig. 4, G to I). Similarly, overexpressing *AtMUTE* in *K. laxiflora* induced additional ACDs rather than paired GC formation like in *A. thaliana* (fig. S7B). Together, this suggested that either species-specific heterodimerization partners, or a diversified genetic program downstream of *MUTE*, underlie the divergent function of *MUTE* in *K. laxiflora*.

### *KlaxMUTE1* induces meristemoid2-specific cell division and identity programs

**Fig. 5.**
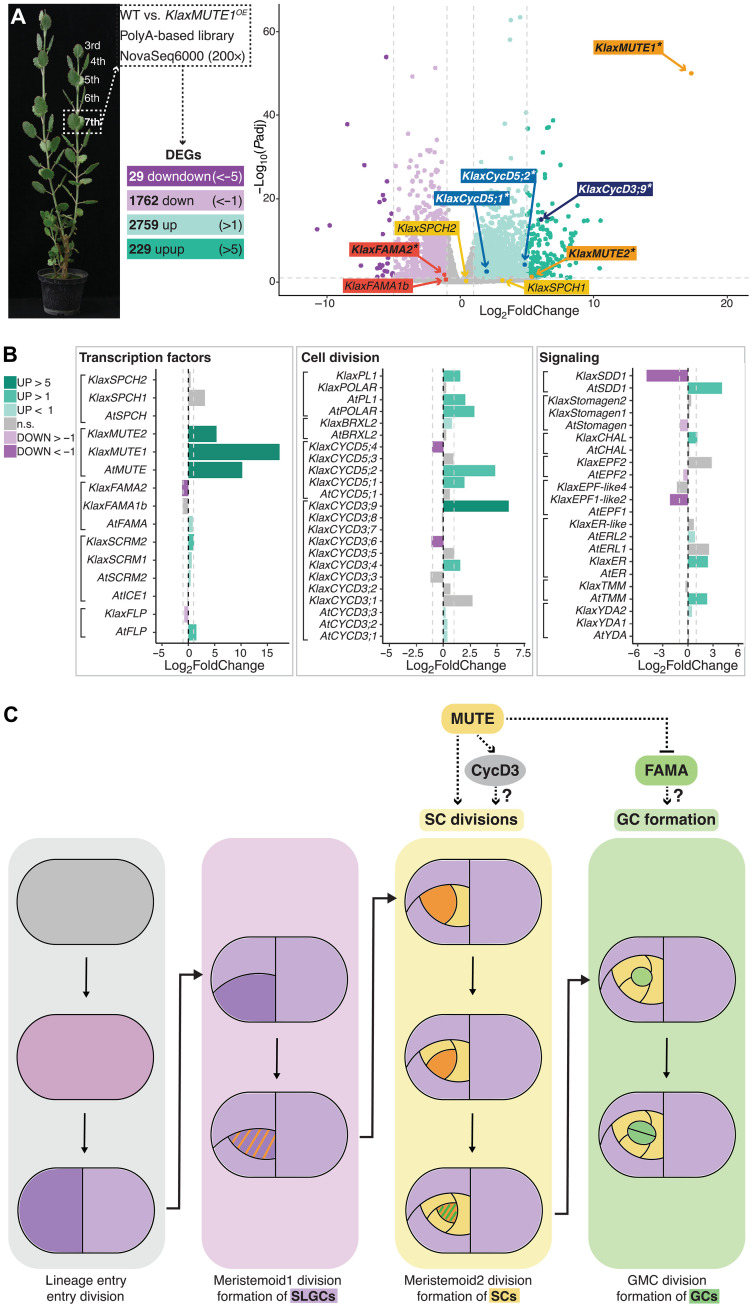
RNA sequencing of *KlaxMUTE1* overexpression line. (**A**) Sampled leaf pairs (LPs), workflow (genotypes used, mRNA enrichment with polyA primers, and sequencing platform as indicated) and volcano plot of up-regulated and down-regulated genes. Number of differentially expressed genes (DEGs) and selected candidate genes are indicated (significant DEGs are printed in bold and with an asterisk). (**B**) Comparison of up-regulation and down-regulation of stomatal development genes by induced *AtMUTE* overexpression in *A. thaliana* (*iMUTE*) (*16*) and *KlaxMUTE1* overexpression in *K. laxiflora*; stomatal transcription factors on the left, cell cycle–related genes in the middle, and signaling factors on the right. (**C**) Model of Crassulaceae SC development; *KlaxMUTEs* drive SC divisions by inducing an asymmetric division program and delaying GC differentiation. Stages are indicated and cell identities color-coded; SLGCs are in lilac, SCs in yellow, GMCs and GCs in green, meristemoid1 in purple, and meristemoid2 in orange. WT, wild type; n.s., not significant.

To determine potential downstream programs activated by *KlaxMUTE1*, we analyzed the transcriptome of mature (seventh) leaves of *KlaxMUTE1^OE^* (Fig. 5A). Compared to mature wild-type leaves, thousands of genes were differentially expressed (2759 up and 1762 down, log_2_FoldChange > 2, *P*adj < 0.1; Fig. 5A and table S3). The most up-regulated gene was the overexpressed *KlaxMUTE1* itself, which also up-regulated its sister gene *KlaxMUTE2* (Fig. 5A and table S3). We then identified homologs of key stomatal regulators described in *A. thaliana* and determined whether some of these were differentially regulated by overexpressing *KlaxMUTE1* compared to induced overexpression of *AtMUTE* in *A. thaliana* (*iMUTE*) using a dataset published previously (Fig. 5B and table S4) (*16*). Notably, core developmental factors associated with GC differentiation like *KlaxFAMA2* and *KlaxFOURLIPS* (*KlaxFLP*) were down-regulated by *KlaxMUTE1^OE^*, which was contrary to *A. thaliana*, where *AtFAMA* and *AtFLP*, and thus GC differentiation, were promoted by induced *AtMUTE* overexpression (Fig. 5B). In addition, two *CycD3* homologs of the substantially amplified *CycD3* gene family (nine genes in *K. laxiflora* and three genes in *A. thaliana*), which are associated with ACDs (*31*), were strongly induced in *KlaxMUTE1^OE^* (Fig. 5B). In *A. thaliana*, *CycD3* genes are bound and activated by *AtSPCH* instead and are posttranslationally inhibited in an AtMUTE-dependent manner during the meristemoid-to-GMC transition (*15*, *16*, *31*–*33*). Many other stomatal regulators behaved similarly in response to *MUTE* overexpression in both *K. laxiflora* and *A. thaliana* (Fig. 5B and tables S3 and S4). In conclusion, some of the downstream genetic modules activated by *MUTE* seem to have diversified in *K. laxiflora* so that GC commitment is delayed and an ACD program required to form SCs is induced.

## DISCUSSION

Much like in grasses, the two *K. laxiflora MUTE* homologs adopted an asymmetric cell division-inducing function to form SCs in eudicot leaf succulents. Considering the substantial evolutionary distance between monocot grasses and eudicot Crassulaceae succulents, and the distinct ontogeny of their perigene and mesogene SCs, respectively, this is a remarkable example of a core stomatal transcription factor taking on a similar role in two distinct plant lineages to form ontogenetically distinct SCs. We propose that *KlaxMUTE1* and *KlaxMUTE2* control an asymmetric division program that extends the rounds of amplifying divisions to form stomatal SCs (Fig. 5C). Furthermore, the weak, transient expression of KlaxMUTE1 at the inferred meristemoid1-to-meristemoid2 transition stage (Fig. 2D) suggested a potential involvement in regulating the transition from meristemoid1 to meristemoid2. In contrast, *AtMUTE* terminates asymmetric divisions, commits meristemoids to GMCs, and promotes their symmetric division (*15*–*17*). The diverse role of the different MUTE proteins, however, seemed not to be encoded within the proteins themselves, as the ACD-promoting versus ACD-inhibiting function could not be transferred from *A. thaliana* to *K. laxiflora* or vice versa. Instead, MUTE proteins activate divergent, species-specific downstream genetic programs to modify developmental programs required to generate GCs in *A. thaliana* and SCs in *K. laxiflora*. This is of little surprise considering that changes to cis-regulatory elements of putative target genes are likely less deleterious and more flexible than mutating the transcription factor itself, and mutations within cis-elements are observed repeatedly as a strategy for generating phenotypic divergence within plants and animals (*34*, *35*). In *A. thaliana*, *AtMUTE* fine-tunes cell cycle and GMC commitment by inducing expression of a GMC-specific CycD5;1 and the GC differentiation factor *AtFAMA* to guarantee a single GMC division and GC differentiation (*16*, *18*). In addition, *AtMUTE*-dependent expression of a cyclin-dependent kinase (CDK) inhibitor (*SIAMESE-RELATED4*) prevents CYCD3;1 and CYCD3;2 from interacting with CDKs, thus preventing ACDs (*15*). In SC-forming *K. laxiflora* on the other hand, *KlaxMUTE1^OE^* induced specific *CycD3* genes to sustain SC-forming ACDs and down-regulated transcription factors like *KlaxFAMA2* and *KlaxFLP* that are associated with GC differentiation and division inhibition in *A. thaliana* (*18*, *36*).

It remains unclear how meristemoid2 divisions are ended and GMCs specified. The absence of mature GCs in *klaxmute1;klaxmute2* could be due to a role of the *KlaxMUTEs* in GMC establishment or a mere consequence of not establishing meristemoid2 identity. Despite the apparent redundancy of *KlaxMUTE1* and *KlaxMUTE2*, their overexpression phenotypes differed slightly. *KlaxMUTE1^OE^* showed lower ploidy and an increase in stomatal density, while stomatal density was slightly decreased in *KlaxMUTE2^OE^*, and the effect on ploidy was weaker. Together, with the strong induction of *KlaxMUTE2* expression in *KlaxMUTE1^OE^* and a slightly weaker and delayed expression of *KlaxMUTE2* translational reporters in stage 3, our results suggest sequential roles for these proteins.

Also, it is unclear how SCs differentiate in *K. laxiflora*. Potentially, SC differentiation is induced directly, postmitotically, as a daughter cell of a meristemoid2 division. Alternatively, the *KlaxMUTEs*’ presence in SCs might be required during the GMC stage (stages 6 and 7), and/or the GMC itself might be involved in the establishment of SC identity through cellular signaling processes. Expression of both *KlaxMUTEs* increased in stage 6 and 7 SCs, which could be due to reinforced cell-autonomous translation, or additional cell-to-cell mobility, as is known for grasses (*11*, *22*). *KlaxMUTEs*’ translational reporter signal in both nucleus and cytoplasm of GMCs was consistent with those of other mobile transcription factors like BdMUTE (*11*) or AtSHR (*37*).

Last, staining for K^+^ within open and closed stomata revealed that the SCs of succulent leaves of *K. laxiflora* are functionally relevant and likely act as stomatal helper cells. In grasses, SCs were shown to act as osmolyte source and sink (*26*), to accommodate GC movement biomechanically by supporting inverse turgor pressurization (*12*, *13*). Furthermore, loss of SCs had a severe negative impact on opening and closing speed when they were genetically ablated in the model grass *Brachypodium distachyon mute* mutant (*11*, *29*). Unlike in grasses, where the perigene SCs stem from a distinct cell lineage to the one that generates GCs, the *klaxmute1;klaxmute2* double mutant aborted both SC and GC formation as they are consecutively formed by the same cell lineage. To demonstrate the physiological role of SCs using genetic ablations, more sophisticated transgene approaches like cell type–specific downregulation through artifical microRNAs (amiRNAs) or well-timed rescue of GMC formation will be required. We speculate, however, that SCs might be pressurized when GCs are closed to minimize stomatal conductance efficiently and prevent daytime transpiration to increase water conservation. The larger total volume of *K. laxiflora* SCs compared to GCs and the fact that SCs are rarely completely devoid of K^+^, even when GCs were forced open, indicate a more prominent role of SCs during stomatal closing.

In conclusion, our work establishes *K. laxiflora* OBG as an experimental model system to study developmental mechanisms in succulents. We found that, much like in grasses, MUTE can be flexibly deployed to facilitate the development of SCs in CAM succulents in the eudicot Crassulaceae family. Our work lays the foundation to manipulate experimentally, and dissect mechanistically, developmental aspects of the water-use efficient CAM leaf anatomy and the inverted daily cycle of stomatal opening and closing that supports CAM. Succulence and (inducible) CAM metabolism are largely ignored yet highly promising traits to engineer water conservation strategies in agricultural systems.

## MATERIALS AND METHODS

### Plant material and growth conditions

*K. laxiflora* accession OBG was used as wild type throughout this study. To grow seedlings on plates, seeds were surface-sterilized using 70% (v/v) ethanol (Thermo Fisher Scientific) for 5 min, washed three times with sterile water, resuspended in 0.1% agarose (BioConcept), and plated on ½ Murashige and Skoog (MS) plates [per liter: 2.2 g of MS medium including Gamborg’s B5 vitamins (Duchefa, M0231), 30 g of sucrose (Thermo Fisher Scientific, BP220), and 8 g of phytoagar (Duchefa, P1003) with pH adjusted to 5.8 using 1 M NaOH]. Plates were grown in a Percival plant growth chamber using diel cycles of 16-hour light/8-hour dark (45 to 50 μmol photosynthetic active radition (PAR) m^−2^ s^−1^, light period temperature = 22°C, dark period temperature = 18°C).

For soil-grown plants, either plants with four to five leaf pairs (LPs; with LP1 being the first visible, youngest LP) and 1-cm-long roots were transplanted from plates to soil, or seeds were directly dispersed in a pot (8 cm in diameter) with seedling substrate (Klasmann-Deilman GmbH, Germany, article #455433). After around 2-week growth under 12-hour light/12-hour dark cycles (300 μmol PAR m^−2^ s^−1^; light period temperature = 24°C, dark period temperature = 18°C), single seedlings were transplanted to bigger pots (12 cm in diameter) containing the following soil: five parts “Landerde” (Ricoter, Switzerland), four parts peat, and one part white sand (hereafter referred as “*Kalanchoë* soil”) and moved to a greenhouse with 16-hour light/8-hour dark cycles (300 μmol PAR m^−2^ s^−1^; light period temperature = 24°C, dark period temperature = 18°C).

*A. thaliana* Col-0 plants for floral dip transformation were grown in a greenhouse with a 16-hour light/8-hour dark cycle (130 to 150 μmol PAR m^−2^ s^−1^; light period temperature = 22°C, dark period temperature = 18°C). Twenty to 30 *Arabidopsis* seedlings were grown in 6-cm pots containing seedling substrate (Klasmann-Deilmann GmbH, Germany) and used for transformation (see below). To grow and select T0 seedlings on plates, seeds were sterilized three times using 70% (v/v) ethanol, poured onto sterile filter paper, dried, and evenly spread on ½ MS plates [per liter: 2.15 g of MS medium (Duchefa, M0221), 30 g of sucrose (Thermo Fisher Scientific, BP220), and 8 g of phytoagar (Duchefa, P1003) with pH adjusted to 5.8 using 1 M NaOH] containing kanamycin (50 mg liter^−1^; CarlRoth, T832).

### Vegetative propagation of *K. laxiflora*

*K. laxiflora* can be grown through stem cuttings or plantlets that emerge on the margins of leaves that were detached from the mother plant. Stem cuttings were cut between LP4 and LP5 and planted into Jiffy pots containing *Kalanchoë* soil. After approximately 2 weeks of root growth in Jiffy pots in a glasshouse under 16-hour light/8-hour dark cycles (300 μmol PAR m^−2^ s^−1^; light period temperature = 24°C, dark period temperature = 18°C), Jiffy pots were transferred to bigger pots (12 cm in diameter) containing *Kalanchoë* soil, and growth was continued in the same conditions.

For plantlet growth, single leaves were detached from the mother plant and placed individually in pots (8 cm in diameter) containing *Kalanchoë* soil. Once the plantlets formed around six LPs, they were transferred individually to Jiffy pots containing *Kalanchoë* soil. Once they reached a stem length of around 10 cm, they were transferred to big pots (12 cm in diameter) containing *Kalanchoë* soil.

### Flower induction in *K. laxiflora*

For flower induction, the main stem of *K. laxiflora* must be at least 10 cm long (around five to six LPs) and grown in long-day conditions (i.e., 16-hour light/8-hour dark) for at least 2 weeks. Plants from a glasshouse grown under 16-hour light/8-hour dark cycles (300 μmol PAR m^−2^ s^−1^; light period temperature = 24°C, dark period temperature = 18°C) were transferred to a Percival plant growth chamber under short-day 8-hour light/16-hour dark cycles (130 to 150 μmol PAR m^−2^ s^−1^; light period temperature = 22°C, dark period temperature = 18°C). Short-day conditions induced flowering after ~3 weeks; however, inflorescence formation was only visible after 4 to 5 weeks of short-day growth. Once flowering was induced, plants were moved to a glasshouse under 16-hour light/8-hour dark cycles (300 μmol PAR m^−2^ s^−1^; light period temperature = 24°C, dark period temperature = 18°C) to expedite flower development.

### Crossing *K. laxiflora*

Around 5 to 6 weeks after inflorescence formation, flowers were mature enough to initiate the crossing process. Flower development in *K. laxiflora* is not synchronized, but different stages of flowers can be found within one inflorescence, resulting in a crossing window of approximately 2 weeks. Flower buds that revealed 5 to 10 mm of petals were carefully opened with forceps, and all eight anthers were carefully removed without injuring the four stigmas. Anthers should look purple and not have dehisced mature pollen already to avoid self-pollination during emasculation. Petals were closed carefully with forceps after emasculation, and emasculated flowers were labeled using tape. Forceps should be cleaned with 70% (v/v) ethanol between each emasculated flower to avoid cross-pollination. Two days after emasculation, flowers were carefully opened using forceps, and mature stigmas were pollinated with mature pollen of the desired genotype. Anthers with mature pollen were obtained from open flowers with white anthers that released pollen.

To produce *klaxmute1;klaxmute2* double mutants, four independent transgenic lines of *klaxmute1* and two independent transgenic lines of *klaxmute2* were used for crossing. *klaxmute1* stigmas were pollinated with *klaxmute2* pollen and vice versa. For crossing the *KlaxMUTEs* translational reporter lines with the plasma membrane marker line, stigmas of four individuals from two independent lines of *KlaxMUTE1:mCitrine-KlaxMUTE1* and one individual of *KlaxMUTE2:mCitrine-KlaxMUTE2* were pollinated with *35S:mCherry-AtPIP1;4* pollen.

### Potassium staining of open and closed stomata

Wild-type *K. laxiflora* OBG plants were grown to the 10 to 12 LP stage. LP6 was collected, and the abaxial side of the epidermis was peeled and float-incubated overnight in an opening or closing buffer [50 mM KCl (Sigma-Aldrich) and 10 mM MES-KOH (Sigma-Aldrich)] with 5 μM fusicoccin (ChemCruz) in the dark or 50 μM ABA (ChemCruz) in the light, respectively. For *A. thaliana*, Col-0 wild-type plants were grown for 8 weeks (16-hour light, 22°C/8-hour dark, 18°C). Mature leaves were collected, and the abaxial epidermis was peeled and incubated overnight in the same way described previously for *K. laxiflora* but incubated in the light with fusicoccin and in the dark with ABA, since *A. thaliana* is a C_3_ plant and therefore keeps its stomata open during the day and closed during the night.

Potassium (K^+^) precipitation was conducted on the basis of (*26*). In short, upon fusicoccin and ABA treatment, leaf peels were placed in 50 mM Ca(NO_3_)_2_ (Thermo Fisher Scientific) in water for 90 s and then in a solution with 0.1 mM Ca(NO_3_)_2_ in water for 30 s. The epidermal peels were then washed with water for 2 min and transferred to a solution of sodium cobaltnitrite [Na_3_Co(NO_2_)_6_; 26.67% (w/v) of Co(NO_3_)_2_ (Sigma-Aldrich) and 46.67% (w/v) of NaNO_2_ (Sigma-Aldrich) in a 13% (v/v) solution of acetic acid (Thermo Fisher Scientific)] in water for 30 min. The samples were washed three times in deionized water for 2 min each and immersed in a solution of 5% (v/v) ammonium sulfide (ChemCruz) to fix the samples. After a final wash in deionized water for 30 s, samples were mounted on slides for visualization and imaged using a Leica DM2000 light-emitting diode (LED) light microscope with a Leica DMC6200 camera.

To quantify in which stomatal cell K^+^ precipitated, three biological replicates with three technical replicates each were analyzed. Each stomatal complex was assigned to one of five categories in *K. laxiflora* and to one of three in *A. thaliana* depending on K^+^ precipitation location. The stomatal complexes were counted fully anonymized by four people and classified, and the percentage for each classification was calculated. A one-way analysis of variance (ANOVA) was performed followed by a 5% Tukey’s post hoc test (α = 0.05) to compare between classes and within treatments and pairwise *t* tests to compare within classes but between treatments.

### Molecular cloning

#### CRISPR-Cas9 constructs

Guide RNA sequences with a high on-target activity score and (almost) no off-targets were selected in Geneious (https://geneious.com/) targeting *KlaxMUTE1* (KlGene012921, priXC24 and priXC25) and *KlaxMUTE2* (KlGene023418, priXC18 and priXC19). To generate the CRISPR-Cas9 constructs, we used an adapted pFASTRK vector from VIB-UGent’s collection (vector ID: 12_01). The binary vector was digested with BsaI (New England Biolabs) and dephosphorylated with Antarctic Phosphatase (BioConcept), and the single-stranded primer guides with appropriate overhangs were annealed, phosphorylated with T4 kinase (New England Biolabs), and ligated into the linearized backbone.

The guide against *KlaxMUTE1* was intentionally designed to potentially also target *KlaxMUTE2*, but only *klaxmute1* single mutant lines were retrieved. For genotyping, leaf genomic DNA was extracted using a modified cetyltrimethylammonium bromide (CTAB) protocol (*20*). The *klaxmute1* mutation was amplified using priXC61 and priYBG4, the *klaxmute2* mutation was amplified using priXC122 and priXC123, and amplicons were Sanger sequenced. The same primers and approach was used to confirm homozygous mutations in *klaxmute1;klaxmute2* double mutant seedlings. All primer sequences can be found in table S1.

#### Reporter and overexpression constructs

All reporter and overexpression constructs were cloned using the GreenGate system (*38*). *KlaxMUTEs* promoter and coding sequence were amplified from *K. laxiflora* OBG wild-type genomic DNA extracted using a modified CTAB protocol (*20*). For creating the entry module *pGGA_KlaxMUTE1pro* and *pGGA_KlaxMUTE2pro*, priXC6 and priXC7 were used to amplify 1313 bp upstream of *KlaxMUTE1*, and priXC59 and priXC11 were used to amplify 2109 bp upstream of *KlaxMUTE2*. The amplicons were digested by BsaI (New England Biolabs) and ligated into the pGGA000 backbone [BsaI digested and dephosphorylated by Antarctic Phosphatase (BioConcept)]. To make the entry modules *pGGC_KlaxMUTE1* (including the STOP codon), primers priXC4 and priXC5 were used to amplify the *KlaxMUTE1* genomic sequence. The fragment was digested by BsaI and ligated into the BsaI-digested and BsaI-dephosphorylated backbone pGGC000 by T4 DNA ligase (New England Biolabs). To create the entry module *pGGC_KlaxMUTE2* (including the STOP codon), two separate fragments were amplified using primers priXC8/priXC12 and priXC13/priXC9 to mutate the BsaI site in the second intron. Both fragments were digested and ligated into the BsaI-digested and BsaI-dephosphorylated backbone pGGC000. All cloned pGGA and pGGC entry vectors were test digested and whole insert Sanger sequenced.

To generate the transcriptional reporter *KlaxMUTE1p:mCitrine-eGFP^nls^*, pGGA_KlaxMUTE1pro, pGGB_mCitrine_IPK_Ala-linker, pGGC_eGFP_NLS (pGGC012), pGGD_Dummy (pGGD002), pGGE_rbscT (pGGE001), and pGGF_NOSpro-KanR (pGGF007) were assembled into the destination vector pGGZ004 by GreenGate cloning. To build the transcriptional reporter *KlaxMUTE2p:mCitrine-eGFP^nls^*, pGGA_KlaxMUTE2pro, pGGB_mCitrine_IPK_Ala-linker, pGGC_eGFP_NLS (pGGC012), pGGD_Dummy (pGGD002), pGGE_rbscT(pGGE001), and pGGF_NOSpro-KanR (pGGF007) were assembled into the destination vector pGGZ004 by GreenGate cloning. To generate the translational reporter *KlaxMUTE1p:mCitrine-KlaxMUTE1*, pGGA_KlaxMUTE1pro, pGGB_mCitrine_IPK_Ala-linker, pGGC-KlaxMUTE1, pGGD-Dummy (pGGD002), pGGE_rbscT (pGGE001), and pGGF_NOSpro-KanR (pGGF007) were assembled into the destination vector pGGZ004 by GreenGate cloning. To build the translational reporter *KlaxMUTE2p:mCitrine-KlaxMUTE2*, pGGA_KlaxMUTE2 pro, pGGB_mCitrine_IPK_Ala-linker, pGGC_KlaxMUTE2, pGGD-Dummy (pGGD002), pGGE_rbscT (pGGE001), and pGGF_NOSpro-KanR (pGGF007) were assembled into the destination vector pGGZ004 by GreenGate cloning. To build the overexpression construct *35S:mCitrine-KlaxMUTE1*, pGGA_35Sp (pGGA004), pGGB_mCitrine_IPK_Ala-linker, pGGC_KlaxMUTE1, pGGD-Dummy (pGGD002), pGGE_rbscT (pGGE001), and pGGF_NOSpro-KanR (pGGF007) were assembled into the destination vector pGGZ004 by GreenGate cloning. To produce the overexpression construct *35S:mCitrine-KlaxMUTE2*, pGGA_35Sp (pGGA004), pGGB_mCitrine_IPK_Ala-linker, pGGC_KlaxMUTE2, pGGD-Dummy (pGGD002), pGGE_rbscT (pGGE001), and pGGF_NOSpro-KanR (pGGF007) were assembled into the destination vector pGGZ004 by GreenGate cloning.

All assembled vectors were test digested and whole-plasmid Sanger sequenced, or the overhang sites were sequenced. The entry modules pGGA000, pGGC000, pGGA004, pGGC012, pGGD002, pGGE001, and pGGF007 were previously described in (*38*). pGGZ004 was described in (*39*). To generate pGGB_mCitrine_IPK_Ala-linker, the mCitrine including the Ala-linker was amplified from the *BdFAMAp:mCitrine-BdFAMA* construct (*40*) using primers priMR454 and priMR455 and generated as described above for promoter and CDS entry clones.

To build the overexpression construct *35S:mCitrine-AtMUTE*(*CDS*): First, primers priTN63 and priTN94 were used to amplify the *AtMUTE* coding sequence from cDNA of *A. thaliana* Col-0 plants, which was synthesized with the PrimeScript RT Master Mix [Perfect Real Time (Takara)] from RNA extracted with the RNeasy Plant Mini Kit (QIAGEN). The entry module pGGC_AtMUTE(CDS) was produced as described above. The binary vector was generated by assembling pGGA_35Sp (pGGA004), pGGB_mCitrine_IPK_Ala-linker, pGGC_AtMUTE(CDS), pGGD-Dummy (pGGD002), pGGE_rbscT (pGGE001), and pGGF_NOSpro-KanR (pGGF007) into the destination vector pGGZ004 using GreenGate cloning.

To clone the plasma membrane marker *35S:mCherry-AtPIP1;4*, pGGA_35Sp (pGGA004), pGGB_mCherry-GSL, pGGC_AtPIP1;4, pGGD-Dummy (pGGD002), pGGE_rbscT (pGGE001), and pGGF_NOSpro-KanR (pGGF007) were assembled into the destination vector pGGZ003 by GreenGate cloning. The pGGB_mCherry-GSL and pGGC_AtPIP1;4 were provided by K. Schumacher and A. Maizel (Centre for Organismal Studies Heidelberg, Germany), respectively. pGGZ003 was previously described in (*38*). All primer sequences can be found in table S1.

### Tissue culture–based transformation of *K. laxiflora* accession OBG 

The stable transformation of *K. laxiflora* OBG followed the protocols described in (*9*, *41*) but adapted as follows. For tissue culture–based transformation, around 10 *K. laxiflora* seedlings per plate were prepared as described above. Around five plates per construct were used for transformation.

To prepare the bacteria cell culture, 4 ml of LB plus the required antibiotics [here: rifampicin (50 mg liter^−1^; Sigma-Aldrich), gentamicin (25 mg liter^−1^; Sigma-Aldrich), and spectinomycin (50 mg liter^−1^; Sigma-Aldrich)] were inoculated with a single colony of the *Agrobacterium tumefaciens* strain GV3101 carrying the construct of interest and incubated overnight at 28°C with rotational shaking at 180 rpm. The overnight culture was pelleted at 3000*g* for 10 min at room temperature in a 50-ml Falcon tube. The pellet was gently resuspended in around 20 ml of MS30 media [per liter: 4.41 g of MS medium including Gamborg’s B5 vitamins (Duchefa, M0231) and 30 g of sucrose (Thermo Fisher Scientific, BP220)], and the optical density at 600 nm was adjusted to 0.1. Last, 40 ml of the MS30 media and bacteria solution was used for transformation. Acetosyringone (Sigma-Aldrich) was added to a final concentration of 100 μM before the tube was wrapped with aluminum foil and incubated with gentle shaking for at least 2 hours at room temperature to induce GV3101 virulence genes that mobilize the transfer DNA.

Meanwhile, the four biggest leaves of the seedlings were harvested into a sterile petri dish using sterile scissors in a sterile laminar flow tissue culture cabinet. Each leaf was cut in two to three pieces using a sterile scalpel with a minimum leaf piece width of around 5 mm. Once all leaf pieces were cut, they were placed into the Falcon tube containing the *A. tumefaciens* cell suspension with the construct of interest. The leaf pieces were incubated in the *A. tumefaciens* cell suspension for approximately 1 hour at room temperature with gentle shaking. Afterward, most of the *A. tumefaciens* solution was removed, and the leaf pieces were placed onto fresh callus induction medium (CIM) plus acetosyringone (Sigma-Aldrich) plates [per liter: 4.41 g of MS medium including Gamborg’s B5 vitamins (Duchefa, M0231), 30 g of sucrose (Thermo Fisher Scientific, BP220), 8 g of phytoagar (Duchefa, P1003), thidiazuron (1 mg liter^−1^; Duchefa, T0916), and indole-3-acetic acid (IAA; 0.2 mg liter^−1^; Duchefa, I0901) with the pH adjusted to 5.7 using 1 M NaOH] plus 100 μM acetosyringone (Sigma-Aldrich) but with no antibiotics for initial cocultivation. Leaf pieces were placed on CIM + acetosyringone plates, wrapped in aluminum foil, and grown for 48 hours at 22°C light period temperature (16 hours) and 18°C dark period temperature (8 hours). After this step, plant tissues were grown in a Percival plant growth cabinet under 16-hour light/8-hour dark cycles (45 to 50 μmol PAR m^−2^ s^−1^; light period temperature = 22°C, dark period temperature = 18°C). Leaf pieces were then transferred to fresh CIM plates with added antibiotics [per liter: 4.41 g of MS medium including Gamborg’s B5 vitamins (Duchefa, M0231), 30 g of sucrose (Thermo Fisher Scientific, BP220), 8 g of phytoagar (Duchefa, P1003), thidiazuron (1 mg liter^−1^; Duchefa, T0916), IAA (0.2 mg liter^−1^; Duchefa, I0901), kanamycin (100 mg/liter^−1^; CarlRoth, T832), and timentin (300 mg liter^−1^; Duchefa, T0190) with the pH adjusted to 5.3 using 1 M NaOH] every 2 weeks until callus formation (approximately 8 to 12 weeks). At each of the first two subculturing iterations, small plantlets that formed on the leaf margins of the leaf pieces were excised and discarded. Once callus was visible on single leaf pieces, those pieces were moved to fresh shoot induction medium (SIM) plates [per liter: 4.41 g of MS medium including Gamborg’s B5 vitamins (Duchefa, M0231), 30 g of sucrose (Thermo Fisher Scientific, BP220), 8 g of phytoagar (Duchefa, P1003), 6-benzylaminopurine (1 mg liter^−1^; Duchefa, B0904), IAA (0.2 mg liter^−1^; Duchefa, I0901), kanamycin (100 mg liter^−1^; CarlRoth, T832), and timentin (300 mg liter^−1^; Duchefa, T0190) with the pH adjusted to 5.1 using 1 M NaOH]. Callus pieces were subcultured every 2 weeks to fresh SIM plates for shoot development (approximately 8 weeks). Shoots that developed from the same callus were numbered and treated as clones. Callus pieces containing several shoots or single shoots were cut into smaller pieces or excised below the stem, respectively, and placed on root induction medium plates [per liter: 4.41 g of MS medium including Gamborg’s B5 vitamins (Duchefa, M0231), 30 g of sucrose (Thermo Fisher Scientific, BP220), 8 g of phytoagar (Duchefa, P1003), kanamycin (50 mg liter^−1^; CarlRoth, T832), and timentin (300 mg liter^−1^; Duchefa, T0190) with the pH adjusted to 5.2 using 1 M NaOH]. Shoots were subcultured every 2 weeks until roots of ~1 cm had developed. Regenerated plants with three to four LPs and a developed root system were transferred to Jiffy pots containing *Kalanchoë* soil and grown in a greenhouse under 16-hour light/8-hour dark cycles (300 μmol PAR m^−2^ s^−1^; light period temperature = 24°C, dark period temperature = 18°C).

### Plant transformation of *A. thaliana*

*A. thaliana* Col-0 wild-type plants were transformed with the *35S:mCitrine-KlaxMUTE1*, *35S:mCitrine-KlaxMUTE2*, and *35S:mCitrine-AtMUTE* constructs, respectively, using the *A. tumefaciens* strain GV3101-based floral dip transformation protocol (*42*).

### Brightfield and confocal microscopy

To image the mature epidermis samples, the abaxial side of LP5 (around 3 cm long) was carefully peeled and moved to a microscopy slide. The epidermal peel was stained with Toluidine Blue O solution {0.2 g of Toluidine Blue O [ChemCruz] in 40 ml of acetate buffer [0.01 g of pectolyase (Duchefa, P8004) in 84.7 ml of 1 M acetic acid (Thermo Fisher Scientific) and 10 ml of distilled water]} for a few seconds and washed with water at least three times until the water on the slide was transparent. The epidermal peel was mounted on a microscopy slide in 50% glycerol (Thermo Fisher Scientific). The samples were imaged on a Leica DM2000LED (Leica Microsystems) using brightfield settings.

To quantify the overexpression lines compared to wild type, three fields of view of three independent individuals per genotype were used. Wild-type–like mature stomatal complexes, mature stomatal complexes with more than three SCs, and the total number of cells per field of view were counted in each image using Fiji (*43*). To calculate stomatal density and total cell density, the number of mature stomatal complexes and the total number of cells were divided by the size of the field of view of 0.198 mm^2^, respectively. The percentage of mature stomatal complexes with more than three SCs was calculated by dividing the number of mature stomatal complexes with more than three SCs by the total number of mature stomata. For plotting and statistical analysis, the values from three fields of view per individual were averaged.

For *klaxmute1;klaxmute2* phenotyping, cotyledons of plate-grown *klaxmute1;klaxmute2* and phenotypic wild-type–like seedlings were carefully collected 14 days after sowing, and the cell membrane of the leaf epidermis was stained with 0.01 mM FM4-64 (Sigma-Aldrich). After staining for 5 min, cotyledons were rinsed with water and mounted on a microscopy slide with water. All samples were imaged with a Leica Stellaris 5 confocal microscope (Leica Microsystems). FM4-64 signal was excited using 549 nm (~10 to 20% intensity) of a white light LED laser running at 85%. Single slice, mid-plane images were taken with a 63× glycerol objective at 1024 by 1024 pixels and a line average of three.

To image the *35Sp:mCitrine-KlaxMUTE1*, *35Sp:mCitrine-KlaxMUTE2*, and *35Sp:mCitrine-AtMUTE* overexpression lines in *A. thaliana*, plate-grown cotyledons of seedlings were collected around 11 days after sowing and stained with FM4-64 as described above. To image the *35Sp:mCitrine-KlaxMUTE1*, *35Sp:mCitrine-KlaxMUTE2*, and *35Sp:mCitrine-AtMUTE* overexpression lines in *K. laxiflora*, the second LP (around 0.5 cm) was collected and stained with FM4-64 as described above. To image overexpression lines in *A. thaliana* and *K .laxiflora*, single-slice, mid-plane images were taken with a 63× glycerol objective at 1024 × 1024 pixels and a line average of three. For FM4-64 excitation, 549 nm (~10 to 20% intensity) of a white light LED laser running at 85% was used. For mCitrine excitation, 515 nm (~10% intensity) of a white light LED laser running at 85% was used.

To image transcriptional and translational reporter lines, 0.5- to 2-cm leaves (mostly second or third LP) were used. To achieve better FM4-64 staining and confocal imaging quality, cuticular wax was removed by gently rubbing the leaf with some water containing hand sanitizer or soap. The leaf was cut next to the mid-vein with a scalpel, and only part of the leaf was used for imaging. For transcriptional reporter lines, leaves were stained with FM4-64 as described above. The translational reporter lines were crossed with the plasma membrane reporter so no staining was needed. To avoid sample damaging, leaf samples were surrounded by four lines of vaseline before mounting in water. The coverslip was carefully placed onto the vaseline having the leaf sit in a water-filled chamber. Single-slice, mid-plane images were taken with a 63× glycerol objective [numerical aperture (NA), 1.3] at 1024 by 1024 pixels and a line average of 3. For FM4-64 or mCherry excitation, 549 nm (~10 to 20% intensity) of a white light LED laser running at 85% was used. For mCitrine excitation, 515 nm (~10% intensity) of a white light LED laser running at 85% was used.

To quantify cell size ratios of induced divisions in *KlaxMUTE1^OE^* and *KlaxMUTE2^OE^*, a 0.5-cm leaf of four different individuals of each of the overexpression lines was imaged and segmented using the software MorphoGraphX (version 1.0 r1280). Each picture represents one replicate. A cell size heatmap for every segmented image was created, and the cell size for every segmented cell was obtained. Cell divisions of recently divided daughter cell pairs were measured, and the postmitotic cell size ratio was calculated (smaller cell/larger cell), resulting in an interval between 0 and 1. The closer to 1, the more symmetrical the division, and conversely, the closer to 0, the more asymmetrical the division.

### Manual time-lapse imaging

The *K. laxiflora* plasma membrane marker line (*35Sp:mCherry-GSL-AtPIP1;4*) and the translational reporter lines for *KlaxMUTE1* (*KlaxMUTE1p:mCit-KlaxMUTE1*; *35Sp:mCherry-GSL-AtPIP1;4*) and *KlaxMUTE2* (*KlaxMUTE2p:mCit-KlaxMUTE2*; *35Sp:mCherry-GSL-AtPIP1;4*) were used for manual time-lapse imaging. The plants were between 10 and 15 cm tall for the stem to be flexible enough for mounting but not too long to risk stem breaking. The pots of the plants were wrapped in cling foil to avoid soil contamination of the microscope. The LP to be imaged was selected (ideally 0.5 to 1 cm), and the second leaf of the pair and any shoot growing above it were removed with clean scissors, as well as other leaves inferring with mounting. The leaf to be imaged was taped to a small plastic petri dish using micropore tape, and part of the edge of the plate was cut out for the stem to lay flat. A spot of lanolin mixed with charcoal was placed on the leaf with a pipet tip, preferably in a relatively flat area between leaf veins. This mark was used as a reference to find the imaged area again. The whole plant was placed on a styrofoam box next to the stage of the Leica Stellaris 5 confocal microscope, and the petri dish with the prepared leaf was placed below the objective. A 40× water dip-in lens (NA, 0.8) was used, and the stage was adjusted to have the lanolin mark right under the center of the objective before placing some water between the sample and the objective. In the brightfield channel, the leaf epidermis was focused, and a notable spot at the edge of the lanolin mark was selected. Then, the channel was switched to fluorescence, and starting at the lanolin landmark, Z-stacks covering the entire depth of the epidermis were taken for four overlapping tiles (with an estimated 10% overlap). For mCherry excitation, 549 nm (~8% intensity) of a white light LED laser running at 85% was used, and the images were taken in a 704 by 704 pixel format, Z-stack step size of 0.41 μm, and a line average of 1. The Z-stacks were converted to three-dimensional objects in Fiji (*43*) and oriented to a “flat” position. The correctly oriented images were saved in .png format and stitched manually using Inkscape [version 1.1 (c68e22387, 2021-05-23)].

When time-lapse imaging, the reporter lines smaller fields of view were imaged without overlapping tiles, and Z-stacks with a step size of 2 μm were taken in a 1024 by 1042 pixel format and a line average of 1. The white light LED laser running at 85% was used for mCitrine excitation (515 nm, ~10% intensity) and mCherry excitation (549 nm, ~8% intensity), and the pinhole aperture was set at 1.7 arbitrary units (AU). Selected slices of the stacks covering the depth of the complex of interest were converted to Z-projects with maximum projection in Fiji (*43*).

### Ploidy analysis

Four adult leaves (around 4 cm) were used per sample, and two samples were prepared for each genotype (wild type, *KlaxMUTE1^OE^*, and *KlaxMUTE2^OE^*). LoBind reaction tubes (Eppendorf) were used for all steps. The abaxial side of the leaf was peeled off with forceps and placed in 250 μl of precooled CyStain UV Precise P Nuclei Extraction Buffer (Sysmex). The peeled epidermis of all four leaves per genotype were pooled in one tube and incubated for 1 min on ice. A 50-μm filter (Sysmex) was pretreated with 250 μl of 1% bovine serum albumin (Sigma-Aldrich) in phosphate-buffered saline [per liter: 8 g of NaCl (Thermo Fisher Scientific), 0.2 g of KCl (Sigma-Aldrich), 1.44 g of Na_2_HPO_4_ (ChemCruz), and 0.24 g of KH_2_PO_4_ (ChemCruz) with pH adjusted to 7.4]. Next, the extracted nuclei were collected by pipetting up and down three times and filtered through the filter. Then, the filter was washed with 500 μl of CyStain UV Precise P Staining Buffer [containing 4′,6-diamidino-2-phenylindole (Sysmex)]. The flow-through was collected in the same tube as the extracted nuclei. The samples were kept on ice and gently mixed before flow cytometry analysis with the BD FACS LSR II system. The data were analyzed with the BD FACSDiva software (version 8.0.1). The counts for both samples per genotype were combined.

### Isolation of high molecular weight total genomic DNA from *K. laxiflora* OBG diploid for genome sequencing

For the isolation of high molecular weight (HMW) total genomic DNA for whole genome sequencing, a single *K. laxiflora* OBG diploid plant was grown and sampled. The single plant sampled was grown from a batch of seed that had passed through three rounds of self-pollination and single seed descent to increase the homozygosity of the genome. The plant was grown to maturity (~30-cm tall) over 6 months in a research glasshouse under 16-hour light/8-hour dark cycles with a minimum temperature in the light period of 20°C. Before leaf sampling for HMW total genomic DNA isolation, the plant was dark-adapted in constant darkness at 15°C for 3 days to ensure all leaf starch had been turned over. LPs 1 and 2 (where LP1 are the first LP that are large enough to handle, either side of the shoot apical meristem) were then collected from multiple shoot tips of the dark-adapted plant, snap frozen in liquid nitrogen, and stored at −80°C until use for genomic DNA isolation.

The frozen, dark-adapted LP1 and LP2 samples were pooled together and ground to fine powder in liquid nitrogen using a pestle and mortar, and then 100 mg of powdered leaf tissue was used for each total genomic DNA isolation using the Cytiva Nucleon PhytoPure 0.1 g kit (Cytiva, RPN8510) according to the manufacturer’s protocol. The quality and quantity of the DNA were analyzed initially using a NanoDrop spectrophotometer, and the average size distribution was checked using agarose gel electrophoresis to ensure that the DNA was intact and of a high average molecular weight, greater than ~30 kb. The HMW total genomic DNA sample was submitted to the University of Liverpool Centre for Genomic Research (CGR) for quality control and library preparation for PacBio HiFi long-read sequencing.

### PacBio library preparation and long-read sequencing

The HMW genomic DNA from *K. laxiflora* OBG diploid was analyzed by the CGR using a Thermo Fisher Scientific Qubit fluorometer for accurate quantification of the double-stranded DNA (dsDNA) and an Agilent fragment analyzer for confirmation that the DNA was of HMW, averaging above ~30 kb.

DNA was then used for PacBio DNA library preparation, and the library was sequenced on the Sequel system using the PacBio SMRT flow cell-8; the library was sequenced on three cells to generate >100-fold genome coverage. The PacBio sequencing chemistry 3.0 was used, and the resulting raw reads were used for genome assembly and annotation, as described below. The PacBio raw reads (BAM files) are available from the National Center for Biotechnology Information (NCBI) Short Read Archive (SRA) under the Bioproject ID PRJEB83678.

### Genome assembly

The raw sequence reads from the three PacBio flow cells were filtered to retain only the reads that were 1 kb or longer, which resulted in 1,744,005 reads that spanned a total of 26.3 Gbp with a median read length of 10,324 bp. The filtered reads were assembled using Canu v1.8 (*44*), yielding an estimated genome size of 260 Mbp, which was consistent with the prediction from flow cytometry analysis of 274 Mb (Flow Cytometry Services, Netherlands). The contigs resulting from the Canu de novo assembly were polished using the PacBio raw reads and the packages pbalign (https://bioconda.github.io/recipes/pbalign/README.html) and Arrow (*45*), with five iterations. The final assembly metrics are summarized in table S5.

Before annotation, the assembled genome was assessed for completeness using BUSCO (Benchmarking Universal Single-Copy Orthologs) v2.0 (*46*) using database species “arabidopsis” and database lineage “embryophyte_odb9.” The results from the BUSCO analysis are summarized in table S6.

### Genome annotation

Gene annotations across the 78 contigs from the Canu assembly were compiled using MAKER (*47*), which uses both multiple sources of published evidence and ab initio prediction to generate gene models.

### Repeat modeling and repeat masking of the genome

Repeat sequences including long terminal repeats (LTRs) and miniature inverted-repeat transposable elements (MITEs) were identified and modeled in the assembled genome using RepeatModeler v1.0.11 (*48*), MiteFinder v1.0.006 (*49*), LTRharvest (*50*), and LTRdigest (*51*) in the GenomeTools package v1.5.11 (*52*). In addition to the genome sequence, various public databases were used in the process, namely, the UniProt UniRef protein database, a eukaryotic tRNA database, and a transposase database.

### Generation of AUGUSTUS ab initio gene models

The ab initio gene predictor AUGUSTUS (*53*) is an integral component of the MAKER pipeline. Here, AUGUSTUS v3.2.3 was used. An initial run of MAKER was performed supported by the following evidence: UniProt/SwissProt proteins, *K. laxiflora* (tetraploid; accession number 1982-6028) 309 v1.1 proteins (from the public database Phytozome), *K. fedtschenkoi* 382 v1.1 proteins [from Phytozome; (*54*)], in-house unpublished proteins from *K. fedtschenkoi* accession “Kew-Glasgow-Liverpool” from Liverpool CGR project LIMS17639, and *K. laxiflora* (tetraploid; accession number 1982-6028) 309 v1.1 transcripts (from Phytozome). MAKER was instructed to predict gene models directly based on transcript and protein evidence mappings (est2genome = 1, protein2genome = 1). This generated predictions that were generally unrefined, but those that were most accurate were used to train AUGUSTUS iteratively. The resulting species-specific hidden Markov model (HMM) was used in the final MAKER run.

### Annotation with MAKER

MAKER v2.31.9 was used to generate a raw set of gene models with the following evidence and parameters. As part of the MAKER run, the software was instructed to repeat-mask the genome using both generic repeat models and the generated genome-specific repeats.

Evidence: UniProt/SwissProt proteins, *K. laxiflora* (tetraploid; accession number 1984-6028) 309 v1.1 proteins (from the public database Phytozome), *K. fedtschenkoi* 382 v1.1 proteins [from Phytozome; (*54*)], in-house unpublished proteins from *K. fedtschenkoi* accession Kew-Glasgow-Liverpool from Liverpool CGR project LIMS17639, and *K. laxiflora* (tetraploid; accession number 1984-6028) 309 v1.1 transcripts (from Phytozome), plus the AUGUSTUS HMM generated as described above. The “Run Arguments” set was as follows: augustus_species = Kl, keep_preds = 1. The AUGUSTUS models were used to predict genes, and all predictions were retained (i.e., whether predictions were supported by provided evidence or not) as these were filtered downstream. This initial annotation with MAKER yielded 30,141 raw gene models.

### Basic functional annotation

Raw protein sequences generated from the raw gene models produced by MAKER were annotated with basic functional information from UnitProt/SwissProt protein alignments that were generated using Basic Local Alignment Search Tool for Proteins (BLASTP).

### Filtering gene models

Raw gene models were filtered to generate a final set of gene models. First, protein sequences derived from the MAKER gene models were annotated using InterProScan v5.31-70.0 (*55*), which detected Pfam domains and Gene Ontology terms. Gene annotations were updated with the InterProScan results. Raw gene models were then filtered and retained if they fulfilled two criteria: (i) They were supported by RNA or protein evidence, or (ii) they had no supporting RNA or protein evidence but were predicted to contain a Pfam domain. This filtering step reduced the final set of predicted genes to 27,768.

### Functional annotation

The final set of 27,768 gene models were annotated with the eggNOG-mapper v1.0.1 (*56*) using the eukaryotic functional database of eggNOG. In addition, the final 27,768 predicted genes were used to reassess genome completeness using BUSCO v2.0 as already described above. Table S7 summarizes the comparison between the initial BUSCO analysis of the assembled genome contigs and the final BUSCO analysis of the 27,768 gene models that were retained after complete annotation and filtering.

### Assessment of duplication based on the final, filtered gene set

In addition to BUSCO analysis providing an estimate of genome/transcriptome completeness and the level of duplication, further analyses were carried out to explore the level of duplication in the genome based on the final filtered gene set. The protein sequences predicted from the final set of 27,768 genes were used as input for cd-hit-est to cluster proteins at 99% identity, and the cluster sizes resulting from this were recorded. Clusters of size 2 or greater would suggest duplication. In addition, clusters were classified as being in the same genomic neighborhood if 25% of genes in the cluster (minimum of 2) were within 50 kb of the genes in the cluster. High proportions of proteins in clusters that included two or more protein sequences could indicate redundancy in the genome assembly or may be caused by true duplicates within the genome. In addition, clusters with two or more genes in the same neighborhood would indicate high levels of local gene duplication. This analysis demonstrated that protein sequences derived from the final set of filtered gene models displayed a very low level of duplication across the *K. laxiflora* OBG diploid genome.

### RNA sequencing of wild type and *KlaxMUTE1^OE^*

For bulk RNA sequencing, the seventh LP was harvested from four wild-type individuals and three independent *KlaxMUTE1^OE^* lines. The samples were snap-frozen in liquid nitrogen, and the total RNA was extracted using the RNeasy Plant Mini Kit with optional on-column deoxyribonuclease digest (QIAGEN). The quantity and quality of the purified total RNA were assessed using a Thermo Fisher Scientific Qubit 4.0 fluorometer with the Qubit RNA BR Assay Kit (Thermo Fisher Scientific, Q10211) and an Advanced Analytical Fragment Analyzer System using a Fragment Analyzer RNA Kit (Agilent, DNF-471), respectively. Sequencing libraries were made with 500 ng of input RNA using a Revelo mRNA-Seq for MagicPrep NGS kit B (Tecan, PN 30186622) according to the Revelo mRNA-Seq for MagicPrep NGS User Guide (Tecan publication number MO1535, v1) with 15 cycles of polymerase chain reaction. The resulting cDNA libraries were evaluated using a Thermo Fisher Scientific Qubit 4.0 fluorometer with the Qubit dsDNA HS Assay Kit (Thermo Fisher Scientific, Q32854) and an Agilent Fragment Analyzer (Agilent) with a HS NGS Fragment Kit (Agilent, DNF-474), respectively. Pooled cDNA libraries were sequenced paired-end using an Illumina NovaSeq 6000 SP Reagent Kit v1.5 [200 cycles (Illumina, 20040719)] on an Illumina NovaSeq 6000 instrument. The quality of the sequencing run was assessed using Illumina Sequencing Analysis Viewer (Illumina version 2.4.7), and all base call files were demultiplexed and converted into FASTQ files using Illumina bcl2fastq conversion software v2.20. The quality control assessments, generation of libraries, and sequencing were conducted by the Next Generation Sequencing Platform, University of Bern. Approximately 23 to 33 million reads were generated per sample (table S3). RNA-seq data have been deposited in Gene Expression Omnibus (GEO) under accession code GSE285473.

The reads were mapped against the *K. laxiflora* OBG genome using HISAT2 and counted using htseq-count on usegalaxy.org. Differentially expressed genes were identified using DESeq2 (*57*). Genes with a *P*adj < 0.1 and a log_2_FoldChange > 1 or log_2_FoldChange < −1 were considered differentially expressed. To compare it to induced *AtMUTE* overexpression (i.e., *iMUTE*), we reanalyzed the dataset from the GEO accession number GSE107018 (*16*) using the above-described pipeline. We identified the *K. laxiflora* OBG homologs of *A. thaliana* stomatal development genes (GO0010374 and own curated list) using local blast against the *K. laxiflora* OBG proteome in Geneious (https://geneious.com/).

## Supplementary Materials

### The PDF file includes:

Figs. S1 to S8 Tables S1, S5 to S7 Legends for tables S2 to S4 Legends for movies S1 and S2
