# Supplementary material for: MUTE drives asymmetric divisions to form stomatal subsidiary cells in Crassulaceae succulents
Source: Sci Adv. 2026 Mar 25;12(13):eaeb8145. doi: 10.1126/sciadv.aeb8145 (PMC13015906; doi:10.1126/sciadv.aeb8145)
Supplement: Supplementary file 1 — Figs. S1 to S8 Tables S1, S5 to S7 Legends for tables S2 to S4 Legends for movies S1 and S2 [file sciadv.aeb8145_sm.pdf]

Science Advances
AAAS

# Supplementary Materials for

## MUTE drives asymmetric divisions to form stomatal subsidiary cells in Crassulaceae succulents

Xin Cheng *et al.*

Corresponding author: Heike Lindner, heike.lindner@unibe.ch; Michael T. Raissig, michael.raissig@unibe.ch

*Sci. Adv.* **12**, eaeb8145 (2026)
DOI: 10.1126/sciadv.aeb8145

**The PDF file includes:**

Figs. S1 to S8
Tables S1, S5 to S7
Legends for tables S2 to S4
Legends for movies S1 and S2

**Other Supplementary Material for this manuscript includes the following:**

Tables S2 to S4
Movies S1 and S2

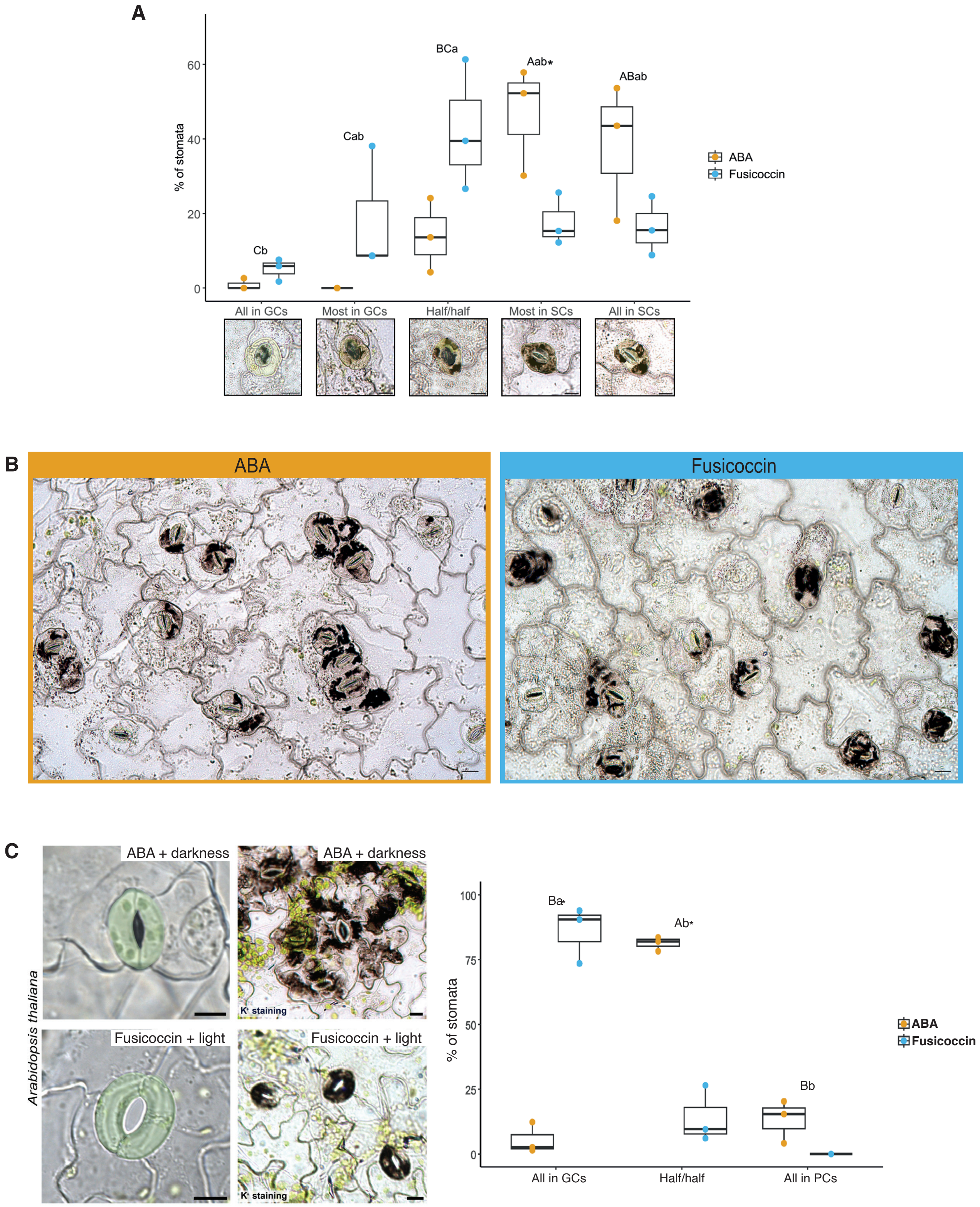


**Fig. S1. Quantification of potassium ($K^+$) levels in open and closed stomata in *K. laxiflora* and *A. thaliana*.**
(A) Statistical analysis of $K^+$ quantification in stomatal complexes of *K. laxiflora* subjected to stomatal opening (Fusicoccin) and closing (ABA) treatment followed by sodium cobaltnitrite treatment for $K^+$ visualization. Each classification is defined by a representative image of the respective class and the stomata were artificially colored in yellow (subsidiary cells) and green (guard cells); 3 individuals and 84-92 stomata were counted per treatment. (B) Overview of an epidermal leaf peel of *K. laxiflora* after ABA treatment (left) and Fusicoccin treatment (right) and $K^+$ staining. Scale bar, 20 µm. (C) ABA- and Fusicoccin-induced stomatal closing and opening of *A. thaliana* epidermal leaf peels (left panels). Sodium cobaltnitrite treatment for $K^+$ precipitation and visualization was performed (middle panels) and quantified (right-most panel). Scale bar, 20 µm. Differences within a treatment were analysed with one-way ANOVA followed by Tukey post-hoc test, and capital letters indicate significance groups for ABA and lowercase letters indicate significance groups for Fusicoccin. Pair-wise t-test was used to test differences between treatments per class and significance is indicated by an asterisk.

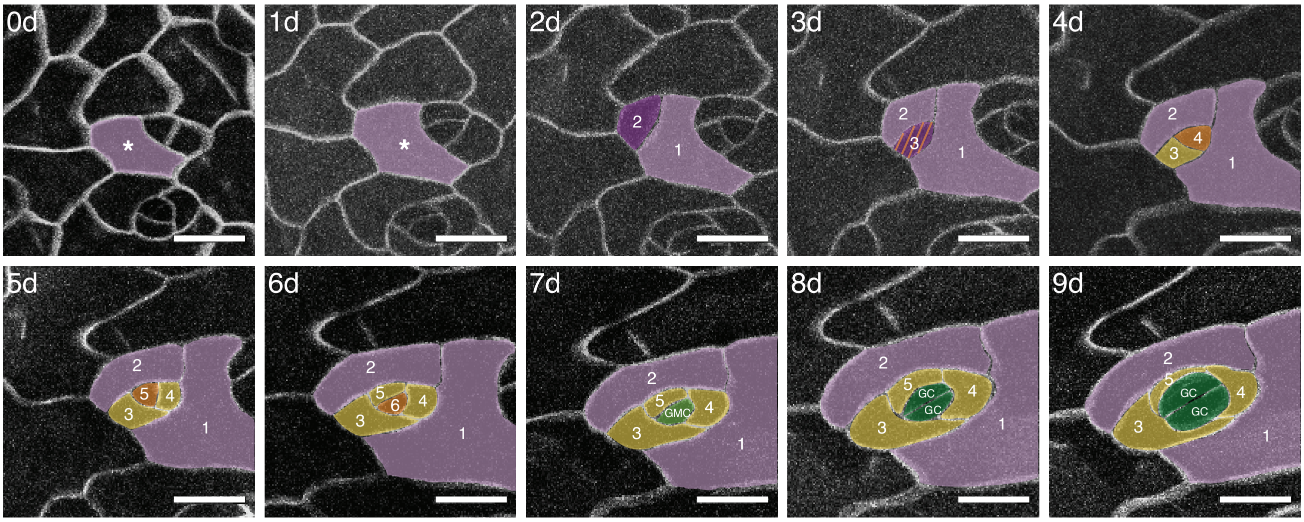


**Fig. S2. 10-day manual time-lapse imaging of plasma membrane marker line *35Sp:mCherry-AtPIP1;4*.** Stomata originating from stomatal lineage ground cells (SLGCs, indicated by an asterisk) omit the entry division and only undergo two meristemoid1, SLGC-producing divisions before transitioning to meristemoid2. Days and cell identities are indicated; SLGCs are in lilac, subsidiary cells (SCs) in yellow, guard mother cells (GMCs) and guard cells (GCs) in green, meristemoid1 in purple, and meristemoid2 in orange. Scale bar, 20 µm.

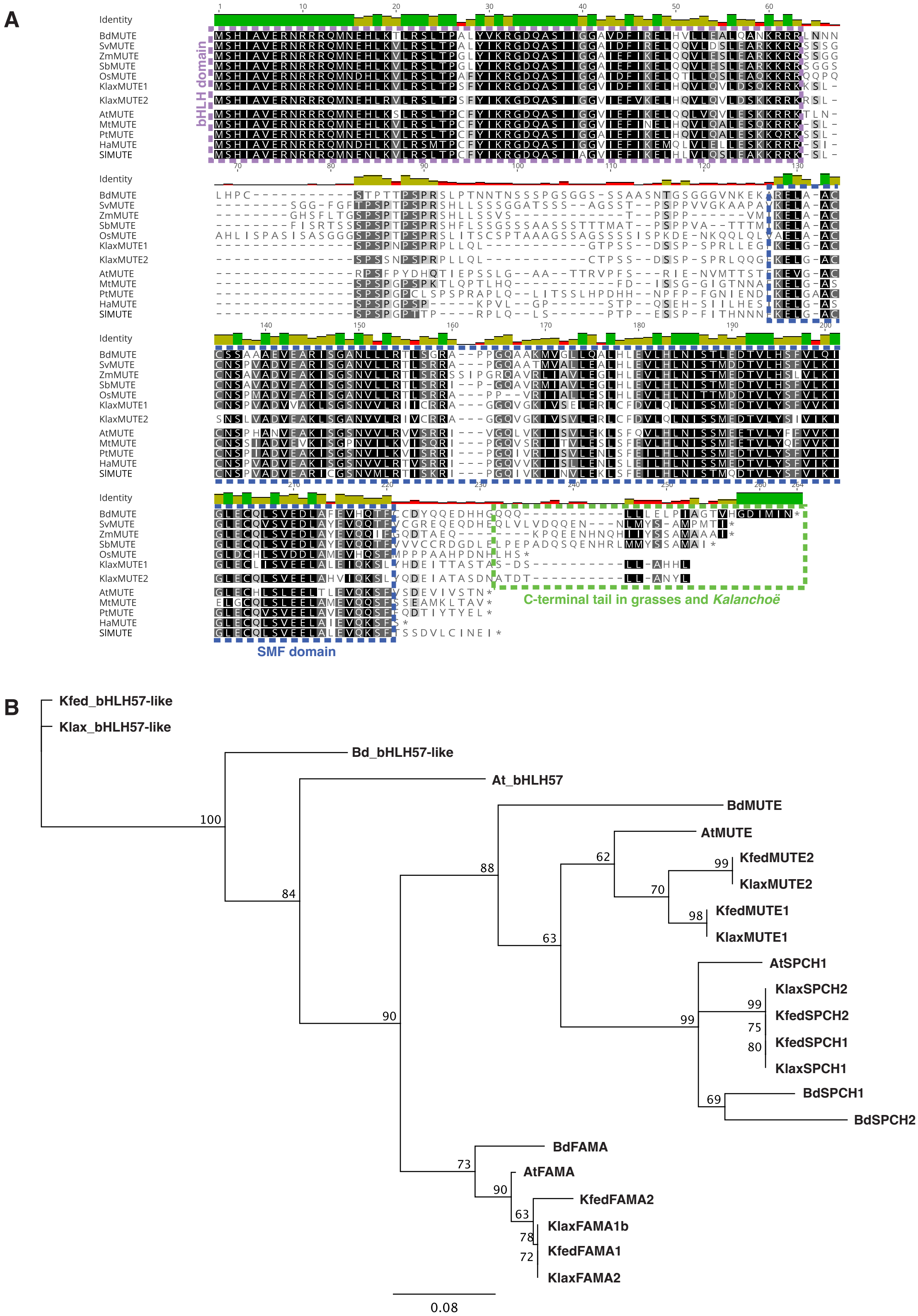


**Fig. S3. MUTE protein alignments and phylogenetic tree of stomatal transcription factors.**

(A) MUTE protein alignment using Clustal Omega. We used five grass and six eudicot species representing the major phylogenetic clades. Grass MUTE protein sequences from *B. distachyon* (Bradi1g18400), *O. sativa* (LOC_Os05g51820), *S. bicolor* (Sobic.009G260200), *Z. mays* (GRMZM2G417164) and *S. viridis* (Sevir.5G150600). Eudicot MUTE protein sequences from *A. thaliana* (At3G06120), *H. annuus* (HanXRQChr12g0362731), *M. truncatula* (Medtr2g022280), *P. trichocarpa* (Potri.008G202900), *S. lycopersicum* (Solyc01g080050) and *K. laxiflora* (KlGene012921 and KlGene023418). Conserved protein domains are indicated with dashed boxes. (B) Phylogenetic tree of stomatal bHLH transcription factors and bHLH57. Protein sequences were aligned and the 63 amino acid-long bHLH domain was isolated. A neighbor-joining tree of the isolated bHLH domains with 100 bootstrap reiterations was generated with standard settings in Geneious. No outgroup was specified. Sequences are from *B. distachyon* (BdMUTE = Bradi1g18400, BdSPCH1 = Bradi1g38650.1, BdSPCH2 = Bradi3g09670.1, BdFAMA = Bradi2g22810.1, Bd_bHLH57-like = Bradi1g71990.2)*;* *A. thaliana* (AtMUTE = At3G06120, AtSPCH =AT5G53210.1, AtFAMA = AT3G24140.1, At_bHLH57 = AT2G46810.1);

**Fig. S3. continued.**

*K. laxiflora* (KlaxMUTE1 = KlGene012921, KlaxMUTE2 = KlGene023418, KlaxSPCH1 = KlGene017537, Klax-SPCH2 = KlGene027982, KlaxFAMA1b = KlGene012763, KlaxFAMA2 = KlOBG012781); *K. fedtschenkoi* (Kfed-MUTE1 =Kaladp0110s0002.1, KfedMUTE2 = Kaladp0058s0199.1, KfedSPCH1 = Kaladp0058s0411.1, KfedSPCH2 = Kaladp0055s0329.1, KfedFAMA1 = Kaladp0081s0065.1, KfedFAMA2 = Kaladp0081s0067.1, Kfed_bHLH57-like = Kaladp0066s0113.1). KlaxFAMA1a was excluded from the analysis. It does not exist in *K. fedtschenkoi* and is not expressed in developing *K. laxiflora* leaves suggesting it to be a pseudogene or wrongly annotated. The Klax_bHLH57-like gene is from the phytozome *K. laxiflora* FTBG accession (Kalaxd.09G089900.1).

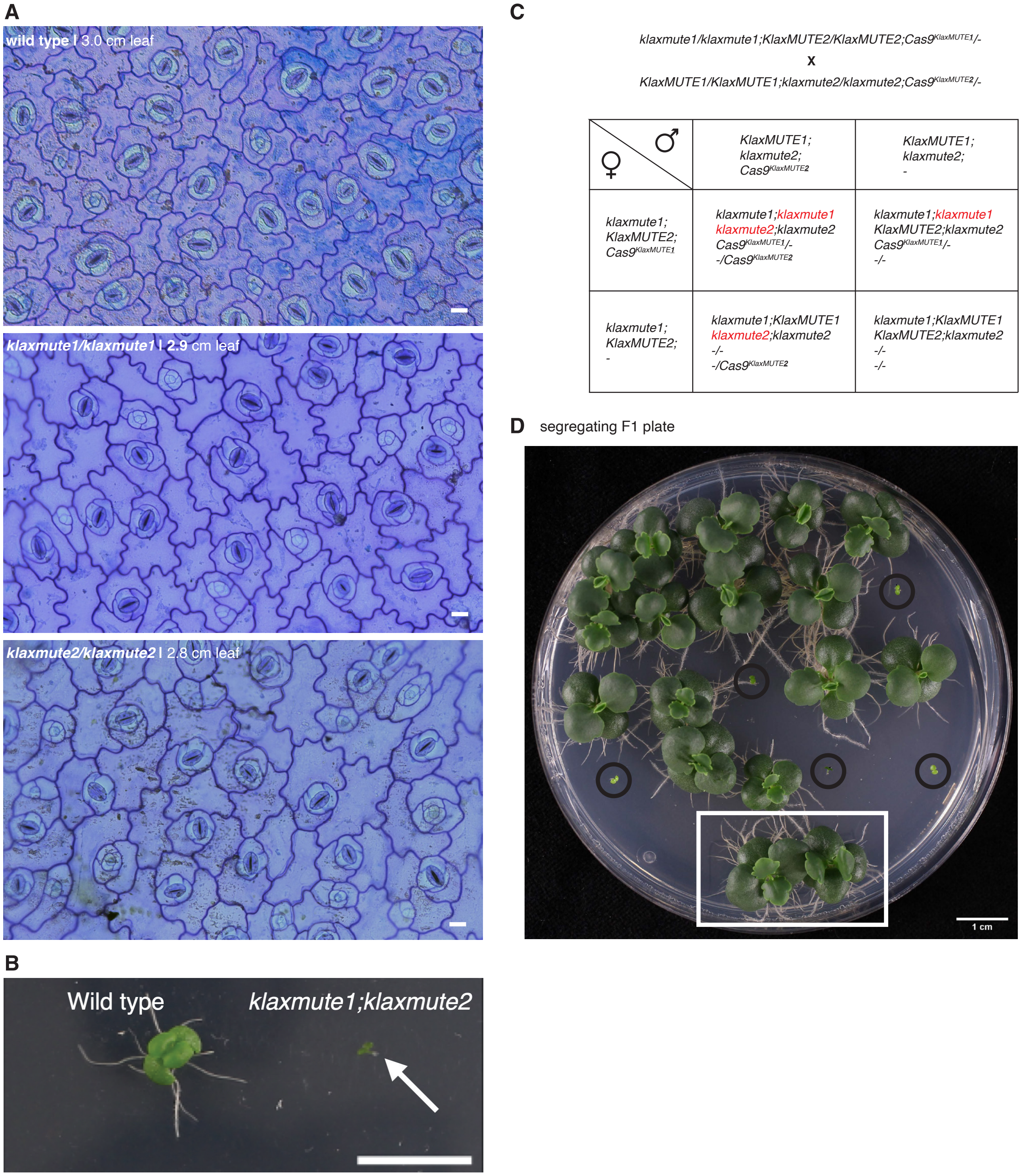


**Fig. S4. *KlaxMUTE1* and *KlaxMUTE2* single mutant phenotypes.**
(A) Brightfield images of Toluidine Blue-stained, abaxial epidermal peels of ~3 cm mature leaves from wild-type (top), *klaxmute1* (middle), and *klaxmute2* (bottom) plants. Scale bar, 20 µm. (B) 25d-old seedlings of wild type and *klaxmute1;klaxmute2* double mutants grown on the same ½ MS plate. Scale bar, 1cm. (C) Punnett square of F1 double mutant screening from crossing *klaxmute1* with *klaxmute2* gene-edited lines that contain an active, hemizygous Cas9 and guideRNA construct. Alleles edited in the F1 generation are printed in red font. (D) 6-week-old segregating F1 seedlings on ½ MS plate. The double mutant, arresting seedlings are encircled in black and the wild-type-like seedlings (double heterozygous or homozygous;heterozygous for *KlaxMUTE1* and/or *KlaxMUTE2*) are not encircled. The white rectangle highlights non-sibling, wild-type plants for comparison. For exact genotypes, see Punnet square in (C). Scale bar, 1cm.

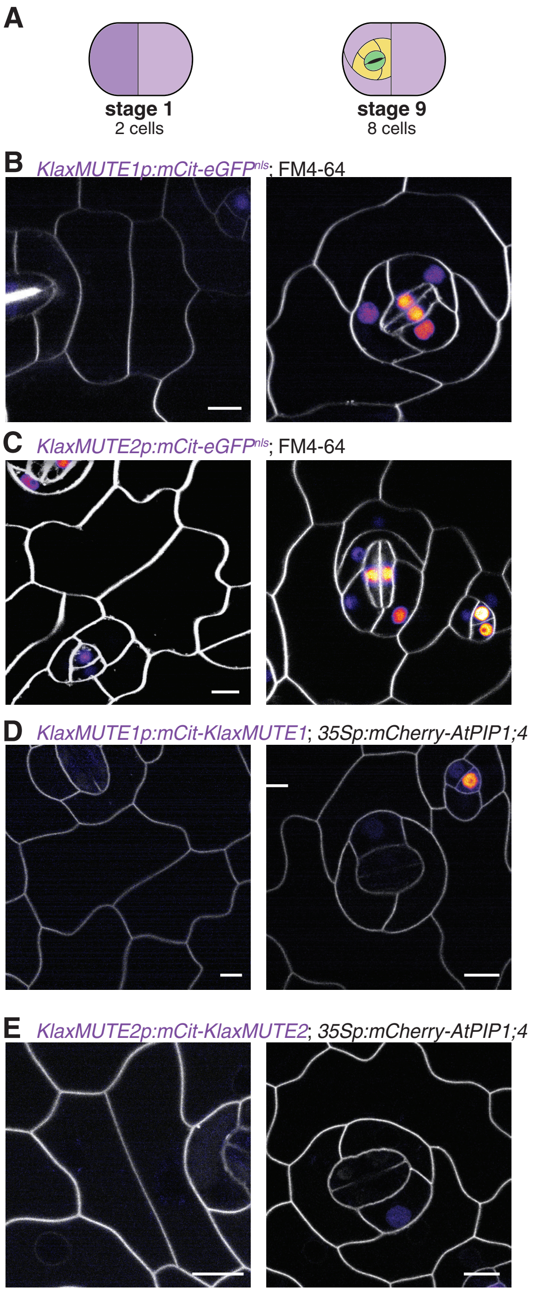


**Fig. S5. Early and mature stages of *KlaxMUTE1* and *KlaxMUTE2* reporter lines.**
(A) Different developmental stages (stage 1 and stage 9) shown below. (B-C) Confocal microscopy images of transcriptional reporters *KlaxMUTE1p:mCitrine-eGFP$^{nls}$* (B) and *KlaxMUTE2p:mCitrine-eGFP$^{nls}$* (C). Cell membrane was stained with FM4-64. (D-E) Confocal microscopy images of translational reporters *KlaxMUTE1p:mCitrine-KlaxMUTE1* (D) and *KlaxMUTE2p:mCitrine-KlaxMUTE2* (E) also expressing the plasma membrane marker *35Sp:mCherry-AtPIP1;4*. YFP signal shown as fire intensity heatmap. Scale bars, 10 µm.

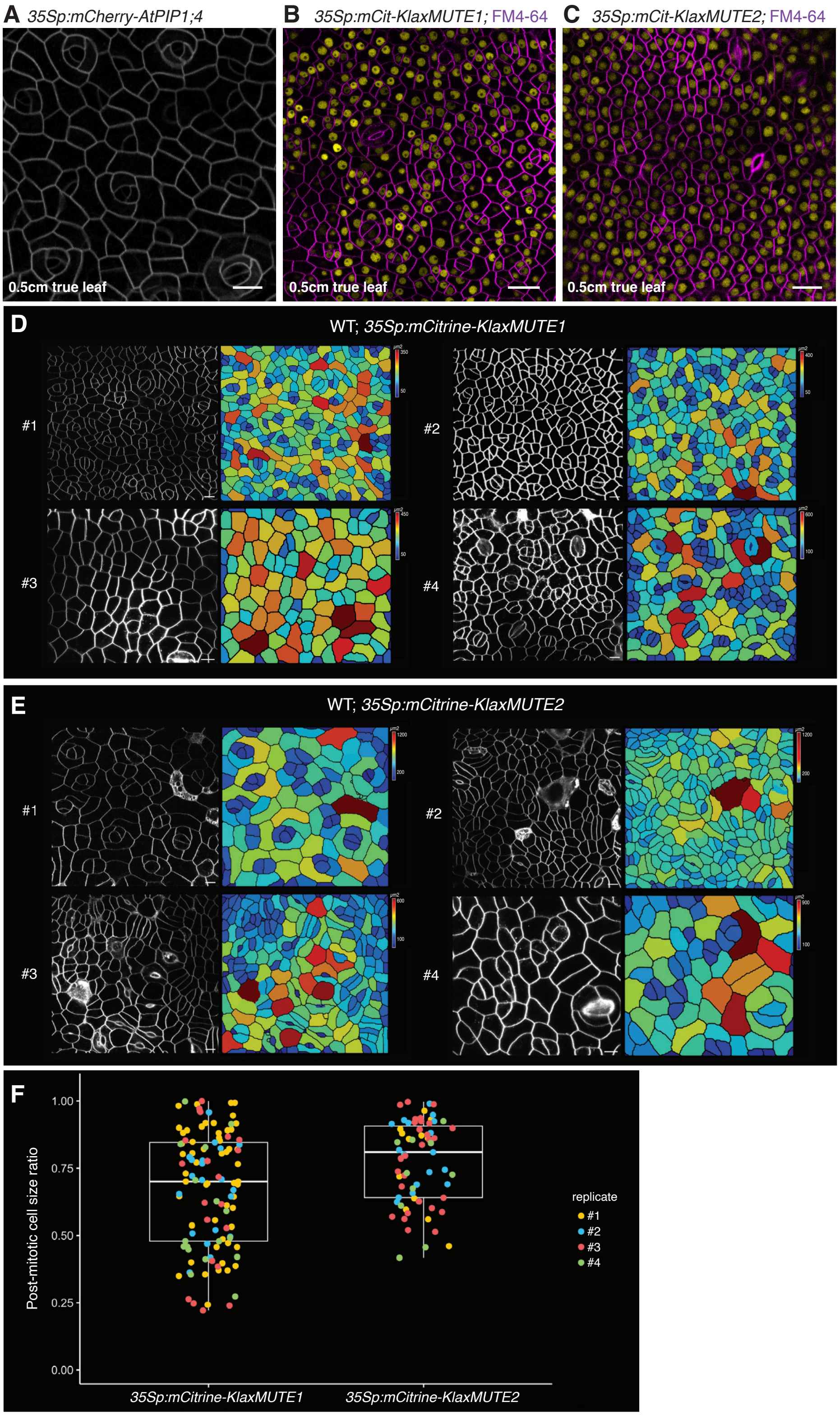


**Fig. S6. Developmental and cellular phenotype of *KlaxMUTEs* overexpression lines in *K. laxiflora*.**
(A-C) Confocal images of the developing abaxial epidermis of ~0.5 cm leaves are shown from *35Sp:mCherry-AtPIP1;4* (in wild type, (A)), *35Sp:mCitrine-KlaxMUTE1* (B), *35Sp:mCitrine-KlaxMUTE2* (C). Cell membrane is stained with FM4-64 in (B) and (C). Scale bar, 20 µm. (D-E) Confocal images and image segmentation of the developing abaxial epidermis of ~0.5 cm leaves of *35Sp:mCitrine-KlaxMUTE1* (D) and *35Sp:mCitrine-KlaxMUTE2* (E). Segmented images and heatmap represents cell size. (F) Quantification of post-division daughter cell size asymmetry ratios. Each dot corresponds to a sister cell pair. Colors correspond to the respective individual images.

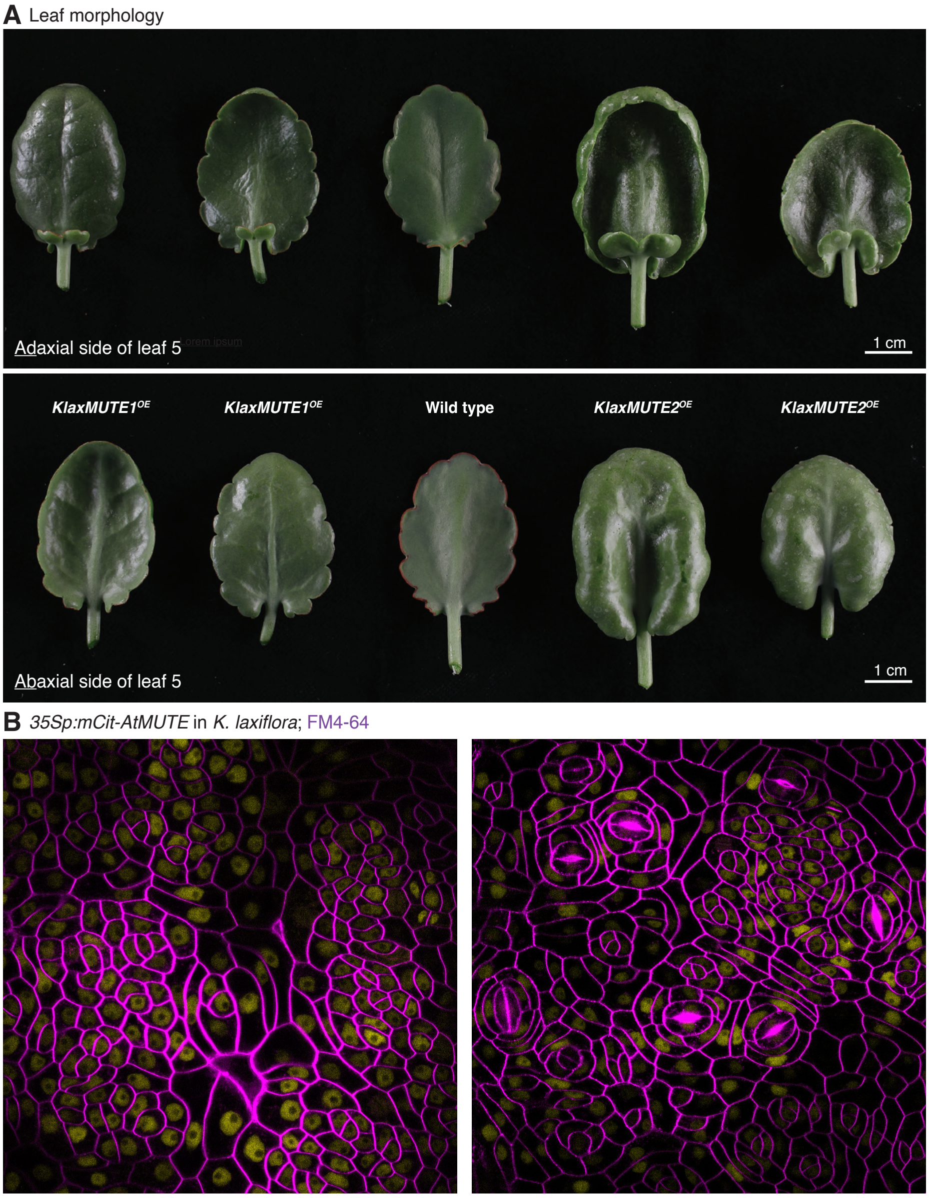


**Fig. S7. Organ-level phenotype of *KlaxMUTE* overexpression and overexpression of *AtMUTE* in *K. laxiflora*.** (A) *K. laxiflora* leaf morphology of adaxial side (top panel) and abaxial side (bottom panel) of leaf 5 of *35Sp:mCitrine-KlaxMUTE1* (first and second leaf), wild type (middle leaf) and *35Sp:mCitrine-KlaxMUTE2* (fourth and fifth leaf). Scale bar, 1 cm. (B) *35Sp:mCitrine-AtMUTE* expression in *K. laxiflora* leaves induces subsidiary cell-like, asymmetric divisions in the epidermis. Scale bar, 20 µm.

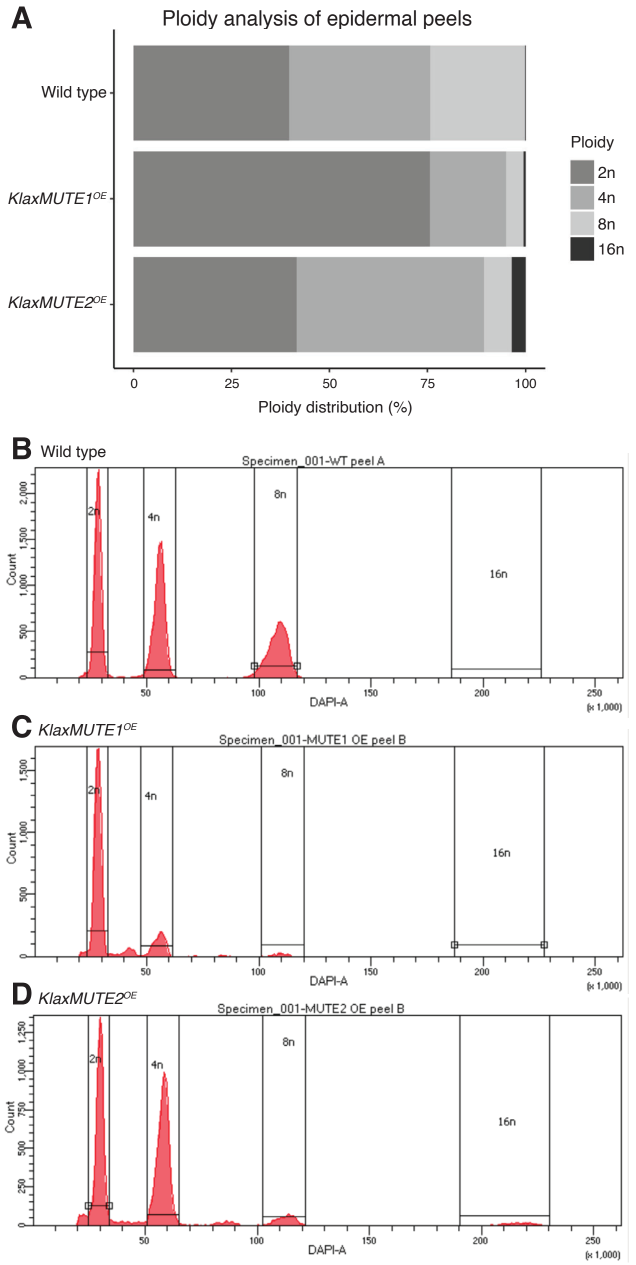


**Fig. S8. Ploidy analysis of leaf epidermal peels of wild type and *KlaxMUTE1* and *KlaxMUTE2* overexpression lines in *K. laxiflora*.**
(A) Quantified ploidy levels from flow cytometry runs of isolated and DAPI-stained nuclei from eight adult leaf epidermal peels per genotype. (B-D) Representative individual flow cytometry runs of wild type (B), *35S:mCitrine-KlaxMUTE1* (C), *35S:mCitrine-KlaxMUTE2* (D) are shown and gating is indicated.

**Table S1.** Primers used in this study.

| Primer name | Primer sequence | Purpose |
|---|---|---|
| priMR454 | AACAGGTCTCAAACAATGGTGAGCAAGGGCGAG | CitrineYFP_FP to clone into pGGB000 |
| priMR455 | AACAGGTCTCAAGCCGGTGAAGGGGGCGGC | CitrineYFP_RP (with pIPK001 linker) to clone into pGGB000 |
| priTN63 | AACAGGTCTCAGGCTCTATGTCTCACATCGCTGTTGA | AtMUTE_FP - bHLH domain |
| priTN94 | AACAGGTCTCACTGATTAATTGGTAGAGACGATCACTTC | AtMUTE_RP |
| priXC4 | AACAGGTCTCA**GGCT**CCATGTCTCACATAGCTGTGGAGC | FP to amplify KlaxMUTE1 KlGene012921 CDS |
| priXC5 | AACAGGTCTCA**CTGA**CTATAAGTGATGAGCAAGCAGTGAG | RP to amplify KlaxMUTE1 KlGene012921 CDS |
| priXC6 | AACAGGTCTCA**ACCT**TACCGTCGACTTTGTGTGAC | FP to amplify KlaxMUTE1 KlGene012921 promoter |
| priXC7 | AACAGGTCTCA**TGTT**GTTTGACTATGGCAGGCAGAA | RP to amplify KlaxMUTE1 KlGene012921 promoter |
| priXC8 | AACAGGTCTCA**GGCT**CCATGTCTCACATAGCGGTGGA | FP to amplify KlaxMUTE2 KlGene023418 CDS |
| priXC9 | AACAGGTCTCA**CTGA**CTATAAGTAGTTTGCAAGCAGTGTG | RP to amplify KlaxMUTE2 KlGene023418 CDS |
| priXC59 | AACAGGTCTCA**ACCT**GTACAACCGCAAGCAGTTTC | FP to amplify KlaxMUTE2 KlGene023418 promoter |
| priXC11 | AACAGGTCTCA**TGTT**GCTCGGCTGAATGGCAG | RP to amplify KlaxMUTE2 KlGene023418 promoter |
| priXC12 | AACAGGTCTCA**CTTA**GTTGGCGAGCTAAAGTGC | RP to mutate BsaI site in KlaxMUTE2 KlGene023418 CDS_used with priXC8 |
| priXC13 | AACAGGTCTCA**TAAG**AGTTATTGATACCAGCAAGT | FP to mutate BsaI site in KlaxMUTE2 KlGene023418 CDS_used with priXC9 |
| priXC18 | attggTGACAGTTTGGCTTCCACGT | FP Guide1 for KlaxMUTE2 |
| priXC19 | aaacACGTGGAAGCCAAACTGTCAc | RP Guide1 for KlaxMUTE2 |
| priXC24 | attgGATCAGGCTTCAATCATAGG | FP Guide2 for both KlaxMUTE homologs |
| priXC25 | aaacCCTATGATTGAAGCCTGATC | RP Guide2 for both KlaxMUTE homologs |
| priXC61 | TTCGATCCTTGACCCCATCT | FP for genotyping KlaxMUTE1 mutation in CRISPR CAS9 mutants |
| priYBG4 | GATGAGCAAGCAGTGAGTCA | RP for genotyping KlaxMUTE1 mutation in CRISPR CAS9 mutants |
| priXC122 | TCAATTTATGCGTCCGTGCT | FP for genotyping KlaxMUTE2 mutation in CRISPR CAS9 mutants |
| priXC123 | GTCTTCCATGCTGCTGATGT | RP for genotyping KlaxMUTE2 mutation in CRISPR CAS9 mutants |

**Table S2.** Count data used for plotting in this study.

*see separate file*

**Table S3.** RNA-sequencing of *K. laxiflora* wild type and *KlaxMUTE1*$^{OE}$. Differentially expressed genes, sequencing statistics and stomatal genes.

*see separate file*

**Table S4.** RNA-sequencing re-analysis of iMUTE in *A. thaliana*. Differentially expressed genes and stomatal genes.

*see separate file*

**Table S5.** Summary of final assembly metrics for the *de novo* PacBio-only genome assembly of *K. laxiflora* OBG diploid.

| | |
|---|---|
| Total number of contigs | 78 |
| Total length of contigs (bp) | 226,713,344 |
| N50 number | 9 |
| N50 length (bp) | 11,021,465 |
| Longest contig (bp) | 20,853,154 |
| % GC | 37 |

**Table S6.** Genome completeness for the *K. laxiflora* OBG diploid genome assessed using BUSCO v2.0.

| | Number of BUSCOs | % of BUSCOs |
|---|---|---|
| Complete BUSCOs | 1298 | 90.1% |
| ……as single copy | 1185 | 82.3% |
| ……as duplicated copies | 113 | 7.8% |
| Fragmented BUSCOs | 35 | 2.4% |
| Missing BUSCOs | 107 | 7.4% |

**Table S7.** Genome completeness comparison of BUSCO analysis of the initial assembled genome contigs in comparison to final filtered set of predicted gene models.

| | % of BUSCOs in assembled genome contigs | % of BUSCOs in final, filtered gene set |
|---|---|---|
| Complete BUSCOs | 90.1% | 89.7% |
| ……as single copy | 82.3% | 81.1% |
| …...as duplicated copies | 7.8% | 8.6% |
| Fragmented BUSCOs | 2.4% | 3.3% |
| Missing BUSCOs | 7.4% | 7.0% |

**Movie S1.** Time-lapsed stomatal development; related to Fig. 1F.

*see separate file*

**Movie S2.** Time-lapsed stomatal development from stomatal lineage ground cell after spacing division; related to Fig. S2.

*see separate file*
